# Ramp sequence may explain synonymous variant association with Alzheimer’s disease in the Paired Immunoglobulin-like Type 2 Receptor Alpha (*PILRA*)

**DOI:** 10.1101/2025.01.06.631528

**Published:** 2025-01-08

**Authors:** Justin B. Miller, J. Anthony Brandon, Lauren M. McKinnon, Hady W. Sabra, Chloe C. Lucido, Josue D. Gonzalez Murcia, Kayla A. Nations, Samuel H. Payne, Mark T.W. Ebbert, John S.K. Kauwe, Perry G. Ridge

**Affiliations:** 1 Department of Pathology and Laboratory Medicine, University of Kentucky, Lexington, KY 40506, USA; 2 Sanders-Brown Center on Aging, University of Kentucky, Lexington, KY 40506, USA; 3 Department of Microbiology, Immunology, and Molecular Genetics, University of Kentucky, Lexington, KY 40506, USA; 4 Division of Biomedical Informatics, Department of Internal Medicine, University of Kentucky, Lexington, KY 40506, USA; 5 Department of Biology, Brigham Young University, Provo, UT 84602; 6 Department of Neuroscience, University of Kentucky, Lexington, KY 40506, USA

**Keywords:** Alzheimer’s disease, ramp sequence, genetic association, codon usage bias, disease association

## Abstract

**BACKGROUND:**

Synonymous variant *NC_000007.14:g.100373690T>C* (*rs2405442:T>C*) in the Paired Immunoglobulin-like Type 2 Receptor Alpha (*PILRA*) gene was previously associated with decreased risk for Alzheimer’s disease (AD) in genome-wide association studies, but its biological impact is largely unknown.

**OBJECTIVE:**

We hypothesized that *rs2405442:T>C* decreases mRNA and protein levels by destroying a ramp of slowly translated codons at the 5’ end of *PILRA*.

**METHODS:**

We assessed *rs2405442:T>C* predicted effects on *PILRA* through quantitative polymerase chain reactions (qPCR) and enzyme-linked immunosorbent assays (ELISA) using Chinese hamster ovary (CHO) cells.

**RESULTS:**

Both mRNA (*P*=1.9184 × 10^−13^) and protein (*P*=0.01296) levels significantly decreased in the mutant versus the wildtype in the direction that we predicted based on destroying a ramp sequence.

**CONCLUSIONS:**

We show that *rs2405442:T>C* alone directly impacts *PILRA* mRNA and protein expression, and ramp sequences may play a role in regulating AD-associated genes without modifying the protein product.

Alzheimer’s disease (AD) is highly heritable, with genetic variants accounting for 58–79% of total dementia risk^1^. Common genetic effects identified through genome-wide association studies (GWAS) implicate approximately 80 genetic risk loci with AD-type dementia^2–9^, yet less is known about which genetic variants drive disease association. Many factors from high-impact diseases in addition to AD (i.e., amyloid plaques and neurofibrillary tangles) contribute to the dementia phenotype^10,11^, and heterogeneity plays a role in several distinct subtypes based on biomarkers^12–14^, genetics^15,16^, imaging^13,17–21^, and impact on daily function^22,23^. Similarly, clinical symptoms of dementia are heterogeneous and based on a progression of amyloid deposition, tau buildup, and neurodegeneration (A/T/N)^24^, with mixed pathologies impacting the speed of cognitive decline^13,17,22,25–29^. While polygenic risk scores (PRS) have recently emerged as a viable tool to aggregate genetic risk across various disease-associated loci so that complex population-specific genetic interactions can be simplified to a single risk score^30,31^, they do not attempt to characterize the biological mechanisms underpinning disease associations, and many associations have yet to be biologically validated. One of those currently unsubstantiated associations is located in the Paired Immunoglobulin-like Type 2 Receptor Alpha (*PILRA*). Here, we biologically assessed the effects of synonymous variant, *NC_000007.14:g.100373690T>C* (*rs2405442:T>C*), and propose that its association with AD is caused by destroying a ramp of slowly-translated codons at the 5’ end of *PILRA*.

Ramp sequences are essential genetic regulatory regions that counterintuitively maximize overall translational efficiency by slowing translation at the 5’ end of genes to evenly space ribosomes, which limits downstream ribosomal collisions and reduces translational errors^32–39^. Specifically, ramp sequences increase mRNA stability and gene expression, especially in genes that have higher ribosome density, higher mRNA levels, and a strong correlation between mRNA and protein expression^38,40^ by reducing ribosome stalling and mRNA degradation via ribosome-associated protein quality control (RQC)^41^. Ramp sequences are phylogenetically conserved^42^, yet differ between human populations^43^ and cell types^44^, which corresponds with population and cell-specific differences in gene expression^38,43,44^. A ramp sequence is present in *PILRA*, which likely helps regulate both protein and mRNA levels within different cell types.

*PILRA* is an inhibitory receptor that regulates immune cells^45^ of the myelomonocytic lineage such as macrophages, dendritic cells, monocytes and monocyte-derived dendritic cells and is highly expressed in the lymph node and neural tissues^46,47^. It functions by negatively regulating neutrophil infiltration and controlling monocyte mobility^45,48^. The innate and adaptive immune responses have been implicated in AD^49^, and gene regulation of *PILRA*-expressing myeloid cells have also associated with AD^50^. AD risk alleles are specifically enriched in active enhancers of myeloid-derived cells that express *PILRA* such as monocytes, macrophages, and microglia, with *PILRA* expression contributing a systemic failure of cell-mediated amyloid-β (Aβ) clearance^51^, which likely contributes to AD onset and progression.

Several studies have found AD-associated variants in *PILRA* to be protective^7,52,53^, yet the protective variant effects are generally attributed to a missense variant, *NC_000007.14:g.100374211A>G* (*rs1859788:A>G*)^53^, which is in high linkage disequilibrium with *rs2405442:T>C*. However, we show that *rs2405442:T>C* alone disrupts the *PILRA* ramp sequence by increasing codon adaptiveness relative to the rest of the transcript, which in turn significantly decreases both mRNA (*P*=1.9184 × 10^−13^) and protein (*P*=0.01296) levels in the direction that we hypothesized based on predicted ramp sequence effects. This study is the first time that ramp sequences have been used to prioritize disease-associated variants for biological validation and offers a likely biological mechanism that can regulate *PILRA* expression without altering the final protein product. Further, these analyses show that synonymous variant *rs2405442:T>C* alone disrupts *PILRA* and may drive association with AD.

## MATERIALS AND METHODS

### Identifying AD-associated genetic variants

We prioritized genetic variants for ramp sequence analyses using the GWAS summary statistics from Jansen, et al.^54^ because they report all single nucleotide polymorphism (SNP) associations with AD that exceeded the genome-wide significance threshold of *P*≤5.0×10^−8^ before accounting for linkage disequilibrium at each locus. While Bellenguez, et al.^2^ report additional genetic associations with AD, we opted to not include their summary statistics in these analyses because they report only variants that are likely independent hits after performing linkage disequilibrium analyses, which greatly reduces the number of reported genetic associations by using p-values to prioritize the independent hits. In some cases, leading variants are chosen based on predicted effects, which would also bias our analyses since variant-level ramp sequence effects have not previously been reported. Additionally, ramp sequences are affected by only exonic coding variants, which are generally rarer than intronic variants in GWAS and may be missed by a clumping approach to choose independent hits. Thus, we decided that the full table of variant-level associations reported by Jansen, et al.^54^ was most appropriate to assess how ramp sequences potentially impact AD. Although 2,357 variants were originally reported^54^, only 51 variants were reported in exonic regions, with only 14 SNPs identified as plausible causal variants based on a fine-mapping model that accounts for *APOE* ε4, roughly corresponding to *P*>~2.0×10^−4^. All computational analyses were limited to those 14 variants.

Ensembl^55^ web queries were conducted in December 2023 to obtain the most severe variant consequence, highest minor allele frequency, Combined Annotation Dependent Depletion (CADD) score^56^, Genomic Evolutionary Rate Profiling (GERP) score^57^, and GRCh38.p14 reference genome chromosome coordinates. RegulomeDB^58^ scores were then queried using the RegulomeDB web interface. All transcript isoforms with GRCh38.p14 reference genome coordinates were downloaded from the National Center for Biotechnology Information (NCBI; https://www.ncbi.nlm.nih.gov/datasets/genome/GCF_000001405.40/) in December 2023.

### Identifying Ramp Sequences

Since ramp sequences are dependent on tissue and cell-specific tRNA pools, we used The Ramp Atlas^44^ to download pre-computed tRNA efficiency values for 62 human tissues included in a consensus dataset derived from the Genotype-Tissue Expression (GTEx)^59^, Functional ANnoTation Of the Mammalian genome (FANTOM5)^60^, and the Human Protein Atlas^47^ databases. An additional file consisting of codon efficiencies from 66 cell types was also downloaded from The Ramp Atlas and used to analyze cell-specific effects on *PILRA* ramp sequences. The relative codon adaptiveness in each tissue or cell type could impact the presence or absence of a ramp sequence by changing where translational bottlenecks occur without altering the DNA sequence. We used ExtRamp^36^ to identify ramp sequences in the reference and mutant sequence for each tissue or cell type individually using the -a option to specify the relative codon adaptiveness for each tissue or cell, which resulted in 256 total ramp sequence calculations for each variant (62 tissues + 66 cell types for both the reference and mutant sequences). By default, ExtRamp identifies ramp sequences based on codon translational efficiencies spanning nine codons, which is roughly the size of a ribosome window^61^. The harmonic mean is then used to determine the translational rate within that window, which is compared to the harmonic mean translational efficiency of the entire gene sequence. True outlier regions that occur at the 5’ end of genes are considered ramp sequences and were reported for each tissue and cell type. All scripts used to identify ramp sequences are available at https://github.com/jmillerlab/PILRA_ramp.

### Biological Assessment of Ramp Sequence Effects in PILRA

Synonymous variant *rs2405442:T>C* in *PILRA* was the only AD-associated variant predicted to destroy a ramp sequence. Since all five *PILRA* isoforms are predicted to have ramp sequences, we opted to use the longest *PILRA* isoform (Ensembl accession: *ENST00000198536.7*; NCBI accession: *NM_013439.3*) to assess *rs2405442:T>C* effects on mRNA and protein levels. DNA sequences for *ENST00000198536.7* (wildtype) and *ENST00000198536.7* containing *rs2405442:T>C* (mutant) were synthesized by GenScript Biotech. A Human c-Myc proto-oncogene (*MYC*) epitope tag, FLAG^®^ epitope tag, and enterokinase cleavage site were attached to the 3’ end of the coding sequences. The reference and mutant sequences with annotated features are depicted in Supplementary Figures S1–S2.

Three independent replicates of quantitative polymerase chain reactions (qPCR) that each contained eight technical replicates were used to assess how synonymous variant, *rs2405442:T>C*, impacted *PILRA* mRNA levels in both the mutant and the wildtype transfected cells. Similarly, three independent sets of eight technical replicates were used to assess how PILRA protein levels differed between the mutant and wildtype using Enzyme-Linked Immunosorbent Assay (ELISA). Detailed instructions for replicating each protocol are described below.

### Transfection of wildtype and mutant transcripts

The wildtype and mutant sequences synthesized by GenScript Biotech were each inserted into separate mammalian expression vectors pCMV6-AN-myc-DDK (ORIGENE, Catalog #PS100016). Transformation protocols were followed as recommended by the manufacturer. In brief, plasmids were transformed into competent DH5a cells, amplified, and purified using the ZymoPURE II Plasmid Maxiprep kit (Catalog #D4203). Purified wildtype and mutant plasmids were then transfected into Chinese Hamster Ovary-K1 (CHO-K1) cells using Lipofectamine^™^ 3000 Transfection Reagent protocol (ThermoFisher, Catalog #15338100). Properly transfected cells were selected using the antibiotic G418 sulfate (ThermoFisher, Catalog #10131035). Transfected cells were grown in F12 media (ThemoFisher, Catalog #11765054) with 10 mg/ml penicillin, 10 μg/ml streptomycin (Gibco, Catalog #21127–022), and 10% FBS (HYCLone Catalog #SH30071.01). Cell media was changed every 48 to 72 hours depending on cell confluency levels. CHO-K1 transfected cells were then used in both the qPCR and ELISA protocols.

### qPCR Protocol

Total RNA was extracted from the mutant and the wildtype CHO-K1 transfected cells using the SPLIT RNA Extraction Kit (Lexogen, Catalog #008) and following the manufacturer guidelines. When the total RNA was purified and ready for quality control, the total RNA concentration was quantified using a NanoDrop spectrophotometer, and the Agilent DNF-471 RNA Kit 15nt (Agilent, Catalog #DNF-471–0500). Reverse transcription of the RNA into complementary DNA (cDNA) was then performed to convert the RNA molecules into their corresponding cDNA sequences using the High-Capacity cDNA Reverse Transcription Kit (Thermo Fisher Scientific, Catalog #4374966). We then performed qPCR to quantify gene expression levels, and cDNA concentration was quantified using the Agilent Femto Pulse. *PILRA* mRNA expression was normalized to total RNA to account for potential differences in qPCR amplification efficiency between tests. We calculated the relative expression of *PILRA* (ΔC_t_) by subtracting the *PILRA* counts (C_t_) from a housekeeping gene, Glyceraldehyde 3-phosphate dehydrogenase (*GAPDH*; i.e., *PILRA* – *GAPDH*). True outliers were then removed to limit potential technical artifacts. Finally, we calculated the fold change in expression (ΔΔC_t_)^62^ using the following equation with the average ΔC_t MUTANT_ and ΔC_t CONTROL_ across all replicates: ΔΔC_t_ = 2-^(ΔCt MUTANT - ΔCt CONTROL)^

### ELISA Protocol

Proteins were extracted from the mutant and the wildtype CHO-K1 transfected cells, and the total protein concentration was quantified using the Pierce^™^ BCA Protein Assay (Thermo Fisher Scientific, Catalog #23225 and 23227) following the manufacturer guidelines. The human PILRA protein concentration was quantitively measured in the mutant and the wildtype CHO-K1 transfected cells using the Human PILR-alpha ELISA Kit (Thermo Fisher Scientific, Catalog #EH368RB) by following the manufacturer guidelines. The concentration of PILRA was then normalized to the total concentration of proteins to account for potential variation between tests.

## RESULTS

### Ramp Sequence Variation Caused by Exonic GWAS Hits

Table 1 lists the 14 credibly causal exonic SNPs spanning 12 genes and 79 isoforms reported by Jansen, et al.^54^. Each SNP was previously associated with AD and is here reported with the following: CADD^56^ score from GRCh38-v1.6; highest MAF reported in Ensembl^55^ from any population in 1000G Phase 3^63^, NHLBI Exome Sequencing Project^64^, and gnomAD^65^; GERP^57^ score from 91_mammals.gerp_conservation_score; RegulomeDB^58^ score; and effect on ramp sequences. Six SNPs (*rs2405442:T>C*, *rs12453:T>C*, *rs7982:A>G*, *rs1859788:A>G*, *rs12459419:C>T*, and *rs2296160:A>G*) are in five genes (*PILRA*, *MS4A6A*, *CR1*, *CLU*, and *CD33*) with ramp sequences. While *rs2405442:T>C*, *rs12453:T>C*, *rs1859788:A>G*, and *rs12459419:C>T* change the ramp sequence length, only *rs2405442:T>C* has a severe impact on ramp sequences by destroying it in at least one tissue or cell type.

### PILRA Ramp Sequence

**Figure 1:**
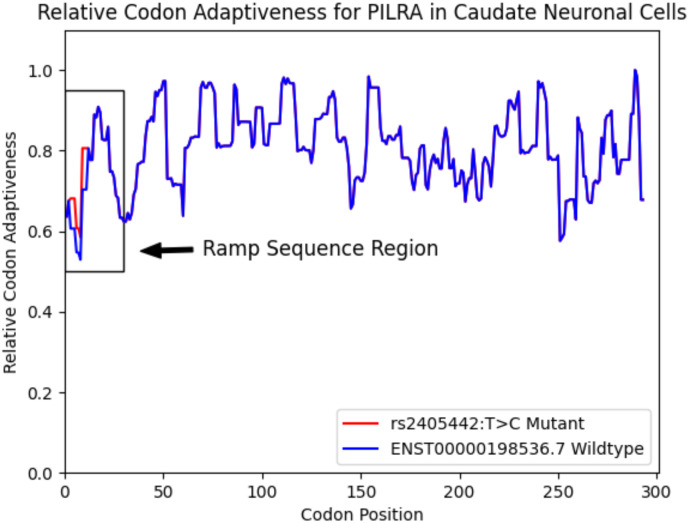
Figure 1 shows the relative codon adaptiveness of the longest PILRA reference isoform and the mutant gene averaged over a nine-codon window in caudate neuronal cells. The mutant gene (*rs2405442:T>C*) has a higher codon adaptiveness at the beginning of the gene sequence compared to the wildtype gene.

Using ExtRamp, we calculated the relative codon adaptiveness of *PILRA* using all four isoforms in GRCh38. We then calculated the relative codon adaptiveness of *PILRA* with synonymous variant *rs2405442:T>C* and found that a ramp sequence is present in the reference isoforms but not the mutant isoforms for all four transcripts. The *PILRA* SNP, *rs2405442:T>C*, increases the regional mean translational efficiency at the 5’ end of *PILRA*, effectively destroying the ramp sequence (see Figure 1).

Using the consensus dataset consisting of gene expression from GTEx, FANTOM5, and the Human Protein Atlas, *PILRA* has a ramp sequence in 26/62 tissues. Using a single-cell dataset from the Human Protein Atlas, we also predicted that ramp sequences occur in *PILRA* in 20/66 cell types. Synonymous variant *rs2405442:T>C* destroyed the ramp sequence in all 46 tissues and cell types that normally contain a ramp sequence. Table 2 lists the 46 tissues or cell types with ramp sequences in *PILRA* that are affected by *rs2405442:T>C* (see Supplementary Table S1 for tissues and cell types without a *PILRA* ramp sequence). Specific neural cells that lost their ramp sequence include cerebellum Purkinje, hippocampus glial, caudate glial, and caudate neuronal cells. Lymphatic tissues and cells that lost their ramp sequence include the dendritic cells, monocytes, appendix lymphoid tissue, lymph node non-germinal center cells, and spleen cells in the red and white pulps.

In addition to ramp sequences, we also evaluated the effects of *rs2405442:T>C* on other codon usage biases such as GC content, codon pairing^66^, codon aversion^67–70^, and codon translational speed. *PILRA* was not previously identified as having splicing quantitative trait loci (sQTLs)^71^, so *rs2405442:T>C* is not predicted to impact splicing. GC content is slightly increased in the mutant, which would normally indicate higher mRNA expression^72^. Additionally, *rs2405442:T>C* affects the twelfth codon in *PILRA*, changing it from an uncommon leucine-encoding codon, TTG, to the most common leucine-encoding codon, CTG, which would normally indicate a higher translational speed since common codons are generally translated faster than rare codons^73^. Similarly, identical codon pairing suggests that *rs2405442:T>C* would increase translational speed^66^ since the mutation increases CTG codon pairing in the transcript from 6 instances in the wildtype to 7 instances in the mutant. Since the synonymous variant does not change the amino acid sequence, co-tRNA codon pairings (i.e., co-occurring amino acid residues)^74^ were not assessed. Based on codon usage biases, the ramp sequence indicates decreased mRNA and protein expression while GC content and codon pairing would suggest increased mRNA and protein expression in the mutant versus the wildtype.

Since synonymous variant *rs2405442:T>C* is the only credible causal synonymous variant with a predicted deleterious effect on a ramp sequence and is highly associated with AD, it was a good candidate for biological validation. We predicted that the destroyed ramp sequence would have an outsized effect due to ribosome-associated protein quality control induced by increased ribosome collisions^41^, and we experimentally validated the predicted effects of *rs2405442:T>C* on *PILRA* mRNA and protein levels with qPCR and ELISA using CHO cells harboring the synonymous variant compared to wildtype cells without the variant.

**Figure 2:**
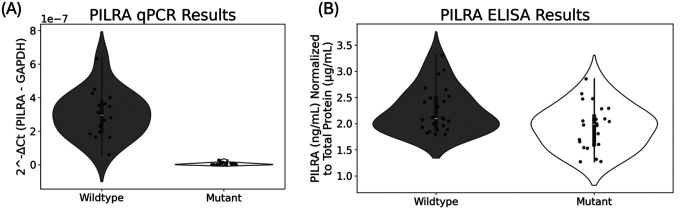
*rs2405442:T>C* effects on mRNA and protein levels in CHO cells harboring the synonymous variant compared to wildtype cells without the variant. (A) shows that *PILRA* mRNA levels are significantly lower in the mutant than the wildtype (P=1.9184 × 10^−13^). Since high C_t_ values show lower expression, we converted the raw C_t_ values to the relative expression by using the formula 2^−Ct^, where C_t_ is the normalized expression of C_*PILRA*_ – C_*GAPDH*_. Two outliers with higher expression from the wildtype were removed, but did not affect the conclusions; (B) shows that *PILRA* protein levels are also significantly lower in the mutant than the wildtype (*P*=0.01296).

Figure 2 shows that mRNA levels are significantly lower in the mutant than the wildtype (*P*=1.9184 × 10^−13^). The fold change in expression (ΔΔC_t_) is ~131x higher in the wildtype than the mutant. Similarly, protein levels are also significantly higher in the wildtype than the mutant (*P*=0.01296), with *PILRA* protein levels being, on average, 1.1635x higher in the wildtype cells than the mutant cells. Although synonymous variant *rs2405442:T>C* has no effect on *PILRA* amino acid residues, it significantly decreases both mRNA and protein levels in the mutant versus the wildtype.

## DISCUSSION

Here, we provide a mechanistic explanation for the association of *rs2405442:T>C* with AD, including experimental validation of its biological effects. This study is the first time that ramp sequences have been used to prioritize disease-associated variants for biological validation. Since reduced *PILRA* inhibitory signaling has previously been shown to induce a protective effect against AD via reduced inhibitory signaling in microglia^53^, it is likely that less *PILRA* expression induced by *rs2405442:T>C* would similarly reduce inhibition of immune cells and result in more efficient cell-mediated clearance of Aβ. Although *rs2405442:T>C* creates a more common codon that increases GC content and codon pairing, which would generally increase mRNA and protein expression, the destroyed ramp sequence outweighs the other codon usage biases producing the observed effects. The destruction of the ramp sequence increases the frequency of ribosomal collisions, leading to stalled proteins and triggering the recruitment of the ribosome-associated protein quality control^41,75^ to degrade aberrant *PILRA* peptides.

*PILRA* gene expression *in vivo* is also likely affected by the distribution of tissue-specific mature tRNA pools. Some evidence suggests that mature tRNA pools change with environmental factors such as aging, stress, and diet, which would also change the relative codon adaptiveness and presence of a ramp sequence^76^. We show that *rs2405442:T>C* alone can significantly affect mRNA and protein levels independent of other genetic variants or changes in the tRNA pool, and further provide a workflow to perform tissue and cell specific computational analyses to investigate how a genetic variant impact gene-specific ramp sequences in different tissues and cells based on tRNA pool availability. Our tissue and cell-specific data show that after acquiring *rs2405442:T>C*, neural tissues such as caudate glial and neuronal cells, cerebellum Purkinje cells, and hippocampus glial cell are predicted to similarly lose their ramp sequence. Many lymphatic and immune-related tissues and cells likewise lose the *PILRA* ramp sequence after acquiring the synonymous variant, including dendritic cells, monocytes, PBMCs, appendix lymphoid tissue, lymph node non-germinal center cells, spleen cells in the red and white pulp, and tonsil non-germinal center cells. Since *PILRA* ramp sequence loss occurs in tissues and cell types known to impact AD, synonymous variant *rs2405442:T>C* in *PILRA* likely protects individuals from AD risk via reduced inhibitory signaling in those tissues and cells.

Synonymous variant *rs2405442:T>C* has a high minor allele frequency (MAF > 0.35^63,65^), indicating that natural decreases in *PILRA* expression induced by this variant are well-tolerated in the general population. Therefore, ramp-mediated therapeutics targeting *rs2405442:T>C* may be viable methods to mitigate risk for AD and increase cell-mediated Aβ clearance without inducing other off-target effects.

Here, we show that a ramp sequence plays a crucial role in *PILRA* gene regulation, and synonymous variant *rs2405442:T>C* alone causes a significant decrease in *PILRA m*RNA and protein levels by disrupting that regulatory mechanism. While synonymous variants are often overlooked in genome-wide association studies, they can significantly alter regulatory biases such as ramp sequences that can directly impact gene expression and protein levels. We outline how to analyze variant effects on ramp sequences, and we present a code repository at https://github.com/jmillerlab/PILRA_ramp to facilitate these types of analyses.

## Supplementary Material

Supplement 1

## Figures and Tables

**Table 1:** Credible Causal Exonic Variant Effects. Credible causality is defined and reported by Jansen, et al.^54^. “Loss of Ramp” indicates that the ramp sequence was destroyed in at least one transcript, while “Ramp Size” indicates that the length of the ramp sequence changed in at least one transcript. “Gene with Ramp” indicates that the SNP was located outside the ramp region, yet the gene has a ramp sequence in at least one transcript in at least one cell or tissue.

SNP	Chromosome/Position	Nearest Gene	Transcripts with Ramp Sequence	Most Severe Variant Effect	Highest MAF	CADD Score	GERP Score	RegulomeDB Score	Most Severe Ramp Effect
**rs2405442:T>C**	7:100373690	*PILRA*	4/4 (100%)	Synonymous	0.50 (T)	4.238	−2.24	1f	Loss of Ramp
**rs12453:T>C**	11:60178272	*MS4A6A*	8/14 (57%)	Synonymous	0.50 (C)	0.578	−4.07	1f	Ramp Size
**rs1859788:A>G**	7:100374211	*PILRA*	4/4 (100%)	Missense	0.50 (A)	12.85	1.01	1f	Ramp Size
**rs12459419:C>T**	19:51225221	*CD33*	2/6 (33%)	Missense	0.48 (T)	14.75	0.06	1f	Ramp Size
**rs7982:A>G**	8:27604964	*CLU*	1/2 (50%)	Missense	0.49 (A)	0.920	−3.07	1f	Gene with Ramp
**rs2296160:A>G**	1:207621975	*CR1*	5/5 (100%)	Missense	0.35 (A)	0.001	−3.64	7	Gene with Ramp
**rs3752241:C>G**	19:1053525	*ABCA7*	0/18 (0%)	Synonymous	0.29 (G)	3.833	−4.46	1f	N/A
**rs117618017:C>T**	15:63277703	*APH1B*	0/3 (0%)	Missense	0.31 (T)	16.39	−1.84	1f	N/A
**rs429358:T>C**	19:44908684	*APOE*	0/5 (0%)	Missense	0.38 (C)	16.65	2.01	1f	N/A
**rs9268480:C>T**	6:32396067	*BTNL2*	0/2 (0%)	Synonymous	0.35 (T)	3.813	−1.07	1f	N/A
**rs1135173:G>A**	2:233146227	*INPP5D*	0/2 (0%)	Synonymous	0.49 (A)	4.311	−3.25	1f	N/A
**rs157581:T>C**	19:44892457	*TOMM40*	0/4 (0%)	Missense	0.50 (C)	14.60	−1.14	1f	N/A
**rs11556505:C>T**	19:44892887	*TOMM40*	0/4 (0%)	Missense	0.18 (T)	6.035	−6.99	5	N/A
**rs75932628:C>T**	6:41161514	*TREM2*	0/2 (0%)	Missense	0.02 (T)	26.1	NA	2b	N/A

**Table 2:** Tissues and cell types that normally have a ramp sequence in *PILRA*. The ramp sequence is universally destroyed in the mutant containing *rs2405442:T>C*.

Tissues with *PILRA* Ramp Sequence	Cell Types with *PILRA* Ramp Sequence
Amygdala	Appendix lymphoid tissue
Cerebral cortex	Caudate glial
Colon	Caudate neuronal
Corpus callosum	Cerebellum Purkinje
Dendritic cells	Cervix uterine glandular
Ductus deferens	Hippocampus glial
Duodenum	Lung pneumocytes
Esophagus	Lymph node nongerminal center
Fallopian tube	Oral mucosa squamous epithelial
Gallbladder	Pancreas islets of Langerhans
Heart muscle	Prostate glandular
Hippocampal formation	Seminal vesicle glandular
Hypothalamus	Skin1 fibroblasts
Monocytes	Skin1 keratinocytes
Olfactory region	Skin1 melanocytes
Pancreas	Soft tissue1 fibroblasts
Retina	Spleen cells in red pulp
Salivary gland	Spleen cells in white pulp
Seminal vesicle	Thyroid gland glandular
Skeletal muscle	Tonsil nongerminal center
Skin	
Small intestine	
Spleen	
Stomach	
Tongue	
Total PBMC	

## Data Availability

All scripts used for these analyses are publicly available at https://github.com/jmillerlab/PILRA_ramp.

## Research in Context:

1. **Systematic review:** Genetic variants identified through genome-wide association studies often lack biological support for their association with Alzheimer’s disease. Although synonymous variant *rs2405442:T>C* in *PILRA* was previously reported as protective against Alzheimer’s disease, its effects have generally been attributed to linkage with missense variant, *rs1859788:A>G*.
2. **Interpretation:** We show that *rs2405442:T>C* alone decreases mRNA and protein levels by destroying a ramp of slowly translated codons at the beginning of *PILRA*. We also show that a ramp sequence is present in *PILRA* and likely regulates mRNA and protein levels, thus offering a plausible biological mechanism explaining *rs2405442:T>C* association with Alzheimer’s disease independent of *rs1859788:A>G*.
3. **Future directions:** We provide the first protocol to evaluate how disease-associated variants impact ramp sequences, which could explain why some genetic variants are reported by genome-wide association studies. Future studies might examine if the ramp sequence could be therapeutically targeted to regulate *PILRA* expression without changing the protein product.
