# Supplementary material for: Ramp sequence may explain synonymous variant association with Alzheimer’s disease in the Paired Immunoglobulin-like Type 2 Receptor Alpha (PILRA)
Source: bioRxiv. 2025 Jan 8:2025.01.06.631528. Preprint. [Version 1] doi: 10.1101/2025.01.06.631528 (PMC11741268; doi:10.1101/2025.01.06.631528)
Supplement: Supplement 1 [file media-1.pdf]

Supplementary Information for
Ramp sequence may explain synonymous variant association with Alzheimer's disease in the Paired Immunoglobulin-like Type 2 Receptor Alpha (*PILRA*)

Justin B. Miller[1,2,3,4,*], J. Anthony Brandon[2], Lauren M. McKinnon[5], Hady W. Sabra[1,2,3,4], Chloe C. Lucido[1,2,3,4], Josue D. Gonzalez Murcia[5], Kayla A. Nations[2], Samuel H. Payne[5], Mark T.W. Ebbert[2,4,6], John S.K. Kauwe[5], Perry G. Ridge[5,*]

[1]Department of Pathology and Laboratory Medicine, University of Kentucky, Lexington, KY 40506, USA
[2]Sanders-Brown Center on Aging, University of Kentucky, Lexington, KY
[3]Department of Microbiology, Immunology, and Molecular Genetics, University of Kentucky, Lexington, KY 40506, USA
[4]Division of Biomedical Informatics, Department of Internal Medicine, University of Kentucky, Lexington, KY 40506, USA
[5]Department of Biology, Brigham Young University, Provo, UT 84602
[6]Department of Neuroscience, University of Kentucky, Lexington, KY 40506, USA

[*]To whom correspondence should be addressed:
Perry Ridge (perry.ridge@byu.edu)
Justin Miller (justin.miller@uky.edu)

Table of Contents

Supplementary Tables *3*

Supplementary Table S1: Tissues and Cell Types Without a Ramp Sequence in *PILRA* 3

Supplementary Figures *5*

Supplementary Figure S1: Wildtype Sequence with Annotated Features 5

Supplementary Figure S2: Mutant Sequence with Annotated Features 13

Supplementary Tables

Supplementary Table S1: Tissues and Cell Types Without a Ramp Sequence in *PILRA*

**Tissues without** *PILRA* Ramp Sequence	**Cell Types without** *PILRA* Ramp Sequence
Adipose tissue	Adrenal gland glandular cells
Adrenal gland	Appendix glandular cells
Appendix	Bone marrow hematopoietic
B-cells	Breast glandular
Basal ganglia	Breast myoepithelial
Bone marrow	Bronchus respiratory epithelial
Breast	Cerebellum glandular layer
Cerebellum	Cerebellum molecular layer
Cervix uterine	Cerebral cortex glial
Endometrium	Cerebral cortex neuronal
Epididymis	Cervix uterine squamous epithelial
Granulocytes	Colon endothelial
Kidney	Colon glandular
Liver	Colon peripheral nerve or ganglion
Lung	Duodenum glandular
Lymph node	Endometrium1 glandular
Midbrain	Endometrium2 glandular
NK-cells	Epididymis glandular
Ovary	Esophagus squamous epithelial
Parathyroid gland	Fallopian tube glandular
Pituitary gland	Gallbladder glandular
Placenta	Heart muscle myocytes
Pons and medulla	Hippocampus neuronal
Prostate	Kidney glomeruli
Rectum	Kidney tubules
Smooth muscle	Liver hepatocytes

Spinal cord	Lung macrophages
Substantia nigra	Lymph node germinal center
T-cells	Nasopharynx respiratory epithelial
Testis	Pancreas exocrine glandular
Thalamus	Parathyroid gland glandular
Thymus	Placenta decidual
Thyroid gland	Placenta trophoblastic
Tonsil	Rectum glandular
Urinary bladder	Salivary gland glandular
Vagina	Skin1 Langerhans
	Skin2 epidermal
	Small intestine glandular
	Stomach1 glandular
	Stomach2 glandular
	Testis cellsinseminiferousducts
	Testis Leydig
	Tonsil germinal center
	Tonsil squamous epithelial
	Urinary bladder urothelial
	Vagina squamous epithelial

Supplementary Figures

Supplementary Figure S1: Wildtype Sequence with Annotated Features

All features and annotations are displayed using SnapGene to illustrate the reference (wildtype) sequence that was transfected into CHO-K1 plasmids. The feature annotations comprise the following seven pages.

Sequence: PILRA_wt_Cterminaltag.dna (Circular / 5786 bp)
Enzymes: Unique 6+ Cutters (50 of 678 total)
Features: 18 total

Unique Cutters **Bold**

```
5'   AACAAAATATTAACGCTTACAATTTCCATTCGCCATTCAGGCTGCGCAACTGTTGGGAAGGGCGATCGGTGCGGGCCTCTTCGCT
...                                                                                          85
3'   TTGTTTTATAATTGCGAATGTTAAAGGTAAGCGGTAAGTCCGACGCGTTGACAACCCTTCCCGCTAGCCACGCCCGGAGAAGCGA
     [f1 ori >]

     ATTACGCCAGCTGGCGAAAGGGGGATGTGCTGCAAGGCGATTAAGTTGGGTAACGCCAGGGTTTTCCCAGTCACGACGTTGTAAA
                                                                                             170
     TAATGCGGTCGACCGCTTTCCCCCTACACGACGTTCCGCTAATTCAACCCATTGCGGTCCCAAAAGGGTCAGTGCTGCAACATTT
                                                                                     [M13 fwd >]

     ACGACGGCCAGTGCCAAGCTGATCTATACATTGAATCAATATTGGCAATTAGCCATATTAGTCATTGGTTATATAGCATAAATCA
                                                                                             255
     TGCTGCCGGTCACGGTTCGACTAGATATGTAACTTAGTTATAACCGTTAATCGGTATAATCAGTAACCAATATATCGTATTTAGT
     [M13 fwd >]

                                                          BsrGI
     ATATTGGCTATTGGCCATTGCATACGTTGTATCTATATCATAATATGTACATTTATATTGGCTCATGTCCAATATGACCGCCATG
                                                                                             340
     TATAACCGATAACCGGTAACGTATGCAACATAGATATAGTATTATACATGTAAATATAACCGAGTACAGGTTATACTGGCGGTAC

                       SpeI      AseI
     TTGACATTGATTATTGACTAGTTATTAATAGTAATCAATTACGGGGTCATTAGTTCATAGCCCATATATGGAGTTCCGCGTTACA
                                                                                             425
     AACTGTAACTAATAACTGATCAATAATTATCATTAGTTAATGCCCCAGTAATCAAGTATCGGGTATATACCTCAAGGCGCAATGT
       [CMV enhancer]

     TAACTTACGGTAAATGGCCCGCCTGGCTGACCGCCCAACGACCCCCGCCCATTGACGTCAATAATGACGTATGTTCCCATAGTAA
                                                                                             510
     ATTGAATGCCATTTACCGGGCGGACCGACTGGCGGGTTGCTGGGGGCGGGTAACTGCAGTTATTACTGCATACAAGGGTATCATT
     [CMV enhancer]

     CGCCAATAGGGACTTTCCATTGACGTCAATGGGTGGAGTATTTACGGTAAACTGCCCACTTGGCAGTACATCAAGTGTATCATAT
                                                                                             595
     GCGGTTATCCCTGAAAGGTAACTGCAGTTACCCACCTCATAAATGCCATTTGACGGGTGAACCGTCATGTAGTTCACATAGTATA
     [CMV enhancer]

     GCCAAGTCCGCCCCCTATTGACGTCAATGACGGTAAATGGCCCGCCTGGCATTATGCCCAGTACATGACCTTACGGGACTTTCCT
                                                                                             680
     CGGTTCAGGCGGGGGATAACTGCAGTTACTGCCATTTACCGGGCGGACCGTAATACGGGTCATGTACTGGAATGCCCTGAAAGGA
     [CMV enhancer]

                      SnaBI
     ACTTGGCAGTACATCTACGTATTAGTCATCGCTATTACCATGGTGATGCGGTTTTGGCAGTACACCAATGGGCGTGGATAGCGGT
                                                                                             765
     TGAACCGTCATGTAGATGCATAATCAGTAGCGATAATGGTACCACTACGCCAAAACCGTCATGTGGTTACCCGCACCTATCGCCA
     [CMV enhancer]                             [CMV promoter >]

     TTGACTCACGGGGATTTCCAAGTCTCCACCCCATTGACGTCAATGGGAGTTTGTTTTGGCACCAAAATCAACGGGACTTTCCAAA
                                                                                             850
     AACTGAGTGCCCCTAAAGGTTCAGAGGTGGGGTAACTGCAGTTACCCTCAAACAAAACCGTGGTTTTAGTTGCCCTGAAAGGTTT
     [CMV promoter >]

     ATGTCGTAATAACCCCGCCCCGTTGACGCAAATGGGCGGTAGGCGTGTACGGTGGGAGGTCTATATAAGCAGAGCTCGTTTAGTG
                                                                                             935
     TACAGCATTATTGGGGCGGGGCAACTGCGTTTACCCGCCATCCGCACATGCCACCCTCCAGATATATTCGTCTCGAGCAAATCAC
     [CMV promoter >]
```

EcoRI SalI AccI BamHI Acc65I KpnI BglII

AACCGTCAGAATTTTGTAATACGACTCACTATAGGGCGGCCGGGAATTCGTCGACTGGATCCGGTACCGAGGAGATCTGCCGCCG 1020
TTGGCAGTCTTAAAACATTATGCTGAGTGATATCCCGCCGGCCCTTAAGCAGCTGACCTAGGCCATGGCTCCTCTAGACGGCGGC

T7 promoter

AsiSI

CGATCGCGCCACCatgggtcggcccctgctgctgcccctactgcccttgctgctgccgccagcatttctgcagcctagtggctcc 1105
GCTAGCGCGGTGGtacccagccggggacgacgacggggatgacgggaacgacgacggcggtcgtaaagacgtcggatcaccgagg

PILRA_wt

Kozak sequence

wt_ttg

Met Gly Arg Pro Leu Leu Leu Pro Leu Leu Pro Leu Leu Leu Pro Pro Ala Phe Leu Gln Pro Ser Gly Ser

acaggatctggtccaagctacctttatggggtcactcaaccaaaacacctctcagcctccatgggtggctctgtggaaatcccct 1190
tgtcctagaccaggttcgatggaaataccccagtgagttggttttgtggagagtcggaggtacccaccgagacacctttagggga

PILRA_wt

Thr Gly Ser Gly Pro Ser Tyr Leu Tyr Gly Val Thr Gln Pro Lys His Leu Ser Ala Ser Met Gly Gly Ser Val Glu Ile Pro

BmgBI

tctccttctattacccctgggagttagccacagctcccgacgtgagaatatcctggagacggggccacttccacaggcagtcctt 1275
agaggaagataatggggaccctcaatcggtgtcgagggctgcactcttataggacctctgccccggtgaaggtgtccgtcaggaa

PILRA_wt

Phe Ser Phe Tyr Tyr Pro Trp Glu Leu Ala Thr Ala Pro Asp Val Arg Ile Ser Trp Arg Arg Gly His Phe His Arg Gln Ser Phe

ctacagcacaaggccgccttccattcacaaggattatgtgaaccggctctttctgaactggacagagggtcagaagagcggcttc 1360
gatgtcgtgttccggcggaaggtaagtgttcctaatacacttggccgagaaagacttgacctgtctcccagtcttctcgccgaag

PILRA_wt

Tyr Ser Thr Arg Pro Pro Ser Ile His Lys Asp Tyr Val Asn Arg Leu Phe Leu Asn Trp Thr Glu Gly Gln Lys Ser Gly Phe

ctcaggatctccaacctgcagaagcaggaccagtctgtgtatttctgccgagttgagctggacacacggagctcagggaggcagc 1445
gagtcctagaggttggacgtcttcgtcctggtcagacacataaagacggctcaactcgacctgtgtgcctcgagtccctccgtcg

PILRA_wt

Leu Arg Ile Ser Asn Leu Gln Lys Gln Asp Gln Ser Val Tyr Phe Cys Arg Val Glu Leu Asp Thr Arg Ser Ser Gly Arg Gln

agtggcagtccatcgaggggaccaaactctccatcacccaggctgtcacgaccaccacccagaggcccagcagcatgactaccac 1530
tcaccgtcaggtagctcccctggtttgagaggtagtgggtccgacagtgctggtggtgggtctccgggtcgtcgtactgatggtg

PILRA_wt

Gln Trp Gln Ser Ile Glu Gly Thr Lys Leu Ser Ile Thr Gln Ala Val Thr Thr Thr Thr Gln Arg Pro Ser Ser Met Thr Thr Thr

ctggaggctcagtagcacaaccaccacaaccggcctcagggtcacacagggcaaacgacgctcagactcttggcacataagtctg 1615
gacctccgagtcatcgtgttggtggtgttggccggagtcccagtgtgtcccgtttgctgcgagtctgagaaccgtgtattcagac

PILRA_wt

Trp Arg Leu Ser Ser Thr Thr Thr Thr Thr Gly Leu Arg Val Thr Gln Gly Lys Arg Arg Ser Asp Ser Trp His Ile Ser Leu

gagactgctgtgggggtggcagtggctgtcactgtgctcggaatcatgatttgggactgatctgcctcctcaggtggaggagaa 1700
ctctgacgacacccccaccgtcaccgacagtgacacgagccttagtactaaaccctgactagacggaggagtccacctcctctt

PILRA_wt

Glu Thr Ala Val Gly Val Ala Val Ala Val Thr Val Leu Gly Ile Met Ile Leu Gly Leu Ile Cys Leu Leu Arg Trp Arg Arg

```
ggaaaggtcagcagcggactaaagccacaaccccagccagggaacccttccaaaacacagaggagccatatgagaatatcaggaa   1785
cctttccagtcgtcgcctgatttcggtgttggggtcggtcccttgggaaggttttgtgtctcctcggtatactcttatagtcctt
PILRA_wt
Arg Lys Gly Gln Gln Arg Thr Lys Ala Thr Thr Pro Ala Arg Glu Pro Phe Gln Asn Thr Glu Glu Pro Tyr Glu Asn Ile Arg Asn

tgaaggacaaaatacagatcccaagctaaatcccaaggatgacggcatcgtctatgcttcccttgccctctccagctccacctca   1870
acttcctgttttatgtctagggttcgatttagggttcctactgccgtagcagatacgaagggaacgggagaggtcgaggtggagt
PILRA_wt
Glu Gly Gln Asn Thr Asp Pro Lys Leu Asn Pro Lys Asp Asp Gly Ile Val Tyr Ala Ser Leu Ala Leu Ser Ser Ser Thr Ser

                                                                        NotI    XhoI PspXI PaeR7I
cccagagcacctcccagccaccgtccccctcaagagcccccagaacgagaccctgtactctgtcttaaaggcGCGGCCGCTCGAGC   1955
gggtctcgtggagggtcggtggcaggggagttctcgggggtcttgctctgggacatgagacagaatttccgCGCCGGCGAGCTCG
PILRA_wt                                                                          1 Glu Myc
Pro Arg Ala Pro Pro Ser His Arg Pro Leu Lys Ser Pro Gln Asn Glu Thr Leu Tyr Ser Val Leu Lys Ala Arg Pro Leu Glu

                                    EcoRV                                   PmeI      FseI
AGAAACTCATCTCAGAAGAGGATCTGGCAGCAAATGATATCCTGGATTACAAGGATGACGACGATAAGGTTTAAACGGCCGGCCG   2040
TCTTTGAGTAGAGTCTTCTCCTAGACCGTCGTTTACTATAGGACCTAATGTTCCTACTGCTGCTATTCCAAATTTGCCGGCCGGC
Gln Lys Leu Ile Ser Glu Glu Asp Leu Ala Ala Asn Asp Ile Leu Asp Tyr Lys Asp Asp Asp Asp Lys Val *
        5               10                  1           5
Myc                         (in frame with Myc)     FLAG
Gln Lys Leu Ile Ser Glu Glu Asp Leu Ala Ala Asn Asp Ile Leu Asp Tyr Lys Asp Asp Asp Asp Lys Val *

 SacII                     XmaI TspMI  SmaI
CGGTCATAGCTGTTTCCTGAACAGATCCCGGGTGGCATCCCTGTGACCCCTCCCCAGTGCCTCTCCTGGCCCTGGAAGTTGCCAC   2125
GCCAGTATCGACAAAGGACTTGTCTAGGGCCCACCGTAGGGACACTGGGGAGGGGTCACGGAGAGGACCGGGACCTTCAACGGTG
M13 rev                        hGH poly(A) signal

  AleI
TCCAGTGCCCACCAGCCTTGTCCTAATAAAATTAAGTTGCATCATTTTGTCTGACTAGGTGTCCTTCTATAATATTATGGGGTGG   2210
AGGTCACGGGTGGTCGGAACAGGATTATTTTAATTCAACGTAGTAAAACAGACTGATCCACAGGAAGATATTATAATACCCCACC
hGH poly(A) signal

                                        BbsI
AGGGGGGTGGTATGGAGCAAGGGGCAAGTTGGGAAGACAACCTGTAGGGCCTGCGGGGTCTATTGGGAACCAAGCTGGAGTGCAG   2295
TCCCCCCACCATACCTCGTTCCCCGTTCAACCCTTCTGTTGGACATCCCGGACGCCCCAGATAACCCTTGGTTCGACCTCACGTC
hGH poly(A) signal

                BsgI                                           BbvCI
TGGCACAATCTTGGCTCACTGCAATCTCCGCCTCCTGGGTTCAAGCGATTCTCCTGCCTCAGCCTCCCGAGTTGTTGGGATTCCA   2380
ACCGTGTTAGAACCGAGTGACGTTAGAGGCGGAGGACCCAAGTTCGCTAAGAGGACGGAGTCGGAGGGCTCAACAACCCTAAGGT
hGH poly(A) signal

                 BlpI
GGCATGCATGACCAGGCTCAGCTAATTTTTGTTTTTTTGGTAGAGACGGGGTTTCACCATATTGGCCAGGCTGGTCTCCAACTCC   2465
CCGTACGTACTGGTCCGAGTCGATTAAAAACAAAAAAACCATCTCTGCCCCAAAGTGGTATAACCGGTCCGACCAGAGGTTGAGG
hGH poly(A) signal
```

BstXI

TAATCTCAGGTGATCTACCCACCTTGGCCTCCCAAATTGCTGGGATTACAGGCGTGAACCACTGCTCCCTTCCCTGTCCTTCTGA 2550
ATTAGAGTCCACTAGATGGGTGGAACCGGAGGGTTTAACGACCCTAATGTCCGCACTTGGTGACGAGGGAAGGGACAGGAAGACT

hGH poly(A) signal

AgeI PflMI

TTTTAAAATAACTATACCAGCAGGAGGACGTCCAGACACAGCATAGGCTACCTGGCCATGCCCAACCGGTGGGACATTTGAGTTG 2635
AAAATTTTATTGATATGGTCGTCCTCCTGCAGGTCTGTGTCGTATCCGATGGACCGGTACGGGTTGGCCACCCTGTAAACTCAAC

hGH poly(A) signal

CTTGCTTGGCACTGTCCTCTCATGCGTTGGGTCCACTCAGTAGATGCCTGTTGAATTGGGTACGCGGCCAGCGGCGAGCGGTATC 2720
GAACGAACCGTGACAGGAGAGTACGCAACCCAGGTGAGTCATCTACGGACAACTTAACCCATGCGCCGGTCGCCGCTCGCCATAG

hGH poly(A) signal

PciI
AflIII

AGCTCACTCAAAGGCGGTAATACGGTTATCCACAGAATCAGGGGATAACGCAGGAAAGAACATGTGAGCAAAAGGCCAGCAAAAG 2805
TCGAGTGAGTTTCCGCCATTATGCCAATAGGTGTCTTAGTCCCCTATTGCGTCCTTTCTTGTACACTCGTTTTCCGGTCGTTTTC

GCCAGGAACCGTAAAAAGGCCGCGTTGCTGGCGTTTTTCCATAGGCTCCGCCCCCCTGACGAGCATCACAAAAATCGACGCTCAA 2890
CGGTCCTTGGCATTTTTCCGGCGCAACGACCGCAAAAAGGTATCCGAGGCGGGGGGACTGCTCGTAGTGTTTTTAGCTGCGAGTT

ori

GTCAGAGGTGGCGAAACCCGACAGGACTATAAAGATACCAGGCGTTTCCCCCTGGAAGCTCCCTCGTGCGCTCTCCTGTTCCGAC 2975
CAGTCTCCACCGCTTTGGGCTGTCCTGATATTTCTATGGTCCGCAAAGGGGGACCTTCGAGGGAGCACGCGAGAGGACAAGGCTG

ori

CCTGCCGCTTACCGGATACCTGTCCGCCTTTCTCCCTTCGGGAAGCGTGGCGCTTTCTCATAGCTCACGCTGTAGGTATCTCAGT 3060
GGACGGCGAATGGCCTATGGACAGGCGGAAAGAGGGAAGCCCTTCGCACCGCGAAAGAGTATCGAGTGCGACATCCATAGAGTCA

ori

ApaLI

TCGGTGTAGGTCGTTCGCTCCAAGCTGGGCTGTGTGCACGAACCCCCCGTTCAGCCCGACCGCTGCGCCTTATCCGGTAACTATC 3145
AGCCACATCCAGCAAGCGAGGTTCGACCCGACACACGTGCTTGGGGGGCAAGTCGGGCTGGCGACGCGGAATAGGCCATTGATAG

ori

GTCTTGAGTCCAACCCGGTAAGACACGACTTATCGCCACTGGCAGCAGCCACTGGTAACAGGATTAGCAGAGCGAGGTATGTAGG 3230
CAGAACTCAGGTTGGGCCATTCTGTGCTGAATAGCGGTGACCGTCGTCGGTGACCATTGTCCTAATCGTCTCGCTCCATACATCC

ori

CGGTGCTACAGAGTTCTTGAAGTGGTGGCCTAACTACGGCTACACTAGAAGAACAGTATTTGGTATCTGCGCTCTGCTGAAGCCA 3315
GCCACGATGTCTCAAGAACTTCACCACCGGATTGATGCCGATGTGATCTTCTTGTCATAAACCATAGACGCGAGACGACTTCGGT

ori

GTTACCTTCGGAAAAAGAGTTGGTAGCTCTTGATCCGGCAAACAAACCACCGCTGGTAGCGGTGGTTTTTTTGTTTGCAAGCAGC 3400
CAATGGAAGCCTTTTTCTCAACCATCGAGAACTAGGCCGTTTGTTTGGTGGCGACCATCGCCACCAAAAAAACAAACGTTCGTCG

ori

AGATTACGCGCAGAAAAAAAGGATCTCAAGAAGATCCTTTGATCTTTTCTACGGGGTCTGACGCTCAGTGGAACGAAAACTCACG 3485
TCTAATGCGCGTCTTTTTTTCCTAGAGTTCTTCTAGGAAACTAGAAAAGATGCCCCAGACTGCGAGTCACCTTGCTTTTGAGTGC

ori

TTAAGGGATTTTGGTCATGAGATTATCAAAAAGGATCTTCACCTAGATCCTTTTAAATTAAAAATGAAGTTTTAAATCAATCTAA 3570
AATTCCCTAAAACCAGTACTCTAATAGTTTTTCCTAGAAGTGGATCTAGGAAAATTTAATTTTTACTTCAAAATTTAGTTAGATT

AGTATATATGAGTAACCTGAGGCTATGGCAGGGCCTGCCGCCCCGACGTTGGCTGCGAGCCCTGGGCCTTCACCCGAACTTGGGG 3655
TCATATATACTCATTGGACTCCGATACCGTCCCGGACGGCGGGGCTGCAACCGACGCTCGGGACCCGGAAGTGGGCTTGAACCCC

GGTGGGGTGGGGAAAAGGAAGAAACGCGGGCGTATTGGCCCCAATGGGGTCTCGGTGGGGTATCGACAGAGTGCCAGCCCTGGGA 3740
CCACCCCACCCCTTTTCCTTCTTTGCGCCCGCATAACCGGGGTTACCCCAGAGCCACCCCATAGCTGTCTCACGGTCGGGACCCT

CCGAACCCCGCGTTTATGAACAAACGACCCAACACCGTGCGTTTTATTCTGTCTTTTTATTGCCGTCATAGCGCGGGTTCCTTCC 3825
GGCTTGGGGCGCAAATACTTGTTTGCTGGGTTGTGGCACGCAAAATAAGACAGAAAAATAACGGCAGTATCGCGCCCAAGGAAGG

HSV TK poly(A) signal

GGTATTGTCTCCTTCCGTGTTTCAGTTAGCCTCCCCCTAGGGTGGGCGAAGAACTCCAGCATGAGATCCCCGCGCTGGAGGATCA 3910
CCATAACAGAGGAAGGCACAAAGTCAATCGGAGGGGGATCCCACCCGCTTCTTGAGGTCGTACTCTAGGGGCGCGACCTCCTAGT

TCCAGCCGGCGTCCCGGAAAACGATTCCGAAGCCCAACCTTTCATAGAAGGCGGCGGTGGAATCGAAATCTCGTGATGGCAGGTT 3995
AGGTCGGCCGCAGGGCCTTTTGCTAAGGCTTCGGGTTGGAAAGTATCTTCCGCCGCCACCTTAGCTTTAGAGCACTACCGTCCAA

BstBI

GGGCGTCGCTTGGTCGGTCATTTCGAACCCCAGAGTCCCGCTCAGAAGAACTCGTCAAGAAGGCGATAGAAGGCGATGCGCTGCG 4080
CCCGCAGCGAACCAGCCAGTAAAGCTTGGGGTCTCAGGGCGAGTCTTCTTGAGCAGTTCTTCCGCTATCTTCCGCTACGCGACGC

265 260 255
* Phe Phe Glu Asp Leu Leu Arg Tyr Phe Ala Ile Arg Gln Ser
NeoR/KanR
* Phe Phe Glu Asp Leu Leu Arg Tyr Phe Ala Ile Arg Gln Ser

AATCGGGAGCGGCGATACCGTAAAGCACGAGGAAGCGGTCAGCCCATTCGCCGCCAAGCTCTTCAGCAATATCACGGGTAGCCAA 4165
TTAGCCCTCGCCGCTATGGCATTTCGTGCTCCTTCGCCAGTCGGGTAAGCGGCGGTTCGAGAAGTCGTTATAGTGCCCATCGGTT

250 245 240 235 230 225
Asp Pro Ala Ala Ile Gly Tyr Leu Val Leu Phe Arg Asp Ala Trp Glu Gly Gly Leu Glu Glu Ala Ile Asp Arg Thr Ala Leu
NeoR/KanR
Asp Pro Ala Ala Ile Gly Tyr Leu Val Leu Phe Arg Asp Ala Trp Glu Gly Gly Leu Glu Glu Ala Ile Asp Arg Thr Ala Leu

CGCTATGTCCTGATAGCGATCCGCCACACCCAGCCGGCCACAGTCGATGAATCCAGAAAAGCGGCCATTTTCCACCATGATATTC 4250
GCGATACAGGACTATCGCTAGGCGGTGTGGGTCGGCCGGTGTCAGCTACTTAGGTCTTTTCGCCGGTAAAAGGTGGTACTATAAG

220 215 210 205 200 195
Ala Ile Asp Gln Tyr Arg Asp Ala Val Gly Leu Arg Gly Cys Asp Ile Phe Gly Ser Phe Arg Gly Asn Glu Val Met Ile Asn
NeoR/KanR
Ala Ile Asp Gln Tyr Arg Asp Ala Val Gly Leu Arg Gly Cys Asp Ile Phe Gly Ser Phe Arg Gly Asn Glu Val Met Ile Asn

GGCAAGCAGGCATCGCCATGGGTCACGACGAGATCCTCGCCGTCGGGCATGCTCGCCTTGAGCCTGGCGAACAGTTCGGCTGGCG 4335
CCGTTCGTCCGTAGCGGTACCCAGTGCTGCTCTAGGAGCGGCAGCCCGTACGAGCGGAACTCGGACCGCTTGTCAAGCCGACCGC

190 185 180 175 170
Pro Leu Cys Ala Asp Gly His Thr Val Val Leu Asp Glu Gly Asp Pro Met Ser Ala Lys Leu Arg Ala Phe Leu Glu Ala Pro Ala
NeoR/KanR
Pro Leu Cys Ala Asp Gly His Thr Val Val Leu Asp Glu Gly Asp Pro Met Ser Ala Lys Leu Arg Ala Phe Leu Glu Ala Pro Ala

CGAGCCCCTGATGCTCTTCGTCCAGATCATCCTGATCGACAAGACCGGCTTCCATCCGAGTACGTGCTCGCTCGATGCGATGTTT 4420
GCTCGGGGACTACGAGAAGCAGGTCTAGTAGGACTAGCTGTTCTGGCCGAAGGTAGGCTCATGCACGAGCGAGCTACGCTACAAA
165 160 155 150 145 140
Leu Gly Gln His Glu Glu Asp Leu Asp Asp Gln Asp Val Leu Gly Ala Glu Met Arg Thr Arg Ala Arg Glu Ile Arg His Lys
NeoR/KanR
Leu Gly Gln His Glu Glu Asp Leu Asp Asp Gln Asp Val Leu Gly Ala Glu Met Arg Thr Arg Ala Arg Glu Ile Arg His Lys

CGCTTGGTGGTCGAATGGGCAGGTAGCCGGATCAAGCGTATGCAGCCGCCGCATTGCATCAGCCATGATGGATACTTTCTCGGCA 4505
GCGAACCACCAGCTTACCCGTCCATCGGCCTAGTTCGCATACGTCGGCGGCGTAACGTAGTCGGTACTACCTATGAAAGAGCCGT
135 130 125 120 115 110
Ala Gln His Asp Phe Pro Cys Thr Ala Pro Asp Leu Thr His Leu Arg Arg Met Ala Asp Ala Met Ile Ser Val Lys Glu Ala
NeoR/KanR
Ala Gln His Asp Phe Pro Cys Thr Ala Pro Asp Leu Thr His Leu Arg Arg Met Ala Asp Ala Met Ile Ser Val Lys Glu Ala

PflFI
Tth111I
GGAGCAAGGTGAGATGACAGGAGATCCTGCCCCGGCACTTCGCCCAATAGCAGCCAGTCCCTTCCCGCTTCAGTGACAACGTCGA 4590
CCTCGTTCCACTCTACTGTCCTCTAGGACGGGGCCGTGAAGCGGGTTATCGTCGGTCAGGGAAGGGCGAAGTCACTGTTGCAGCT
105 100 95 90 85
Pro Ala Leu His Ser Ser Leu Leu Asp Gln Gly Pro Val Glu Gly Leu Leu Leu Trp Asp Arg Gly Ala Glu Thr Val Val Asp Leu
NeoR/KanR
Pro Ala Leu His Ser Ser Leu Leu Asp Gln Gly Pro Val Glu Gly Leu Leu Leu Trp Asp Arg Gly Ala Glu Thr Val Val Asp Leu

GCACAGCTGCGCAAGGAACGCCCGTCGTGGCCAGCCACGATAGCCGCGCTGCCTCGTCTTGCAGTTCATTCAGGGCACCGGACAG 4675
CGTGTCGACGCGTTCCTTGCGGGCAGCACCGGTCGGTGCTATCGGCGCGACGGAGCAGAACGTCAAGTAAGTCCCGTGGCCTGTC
80 75 70 65 60 55
Val Ala Ala Cys Pro Val Gly Thr Thr Ala Leu Trp Ser Leu Arg Ala Ala Glu Asp Gln Leu Glu Asn Leu Ala Gly Ser Leu
NeoR/KanR
Val Ala Ala Cys Pro Val Gly Thr Thr Ala Leu Trp Ser Leu Arg Ala Ala Glu Asp Gln Leu Glu Asn Leu Ala Gly Ser Leu

KasI NarI SfoI PluTI
GTCGGTCTTGACAAAAAGAACCGGGCGCCCCTGCGCTGACAGCCGGAACACGGCGGCATCAGAGCAGCCGATTGTCTGTTGTGCC 4760
CAGCCAGAACTGTTTTTCTTGGCCCGCGGGGACGCGACTGTCGGCCTTGTGCCGCCGTAGTCTCGTCGGCTAACAGACAACACGG
50 45 40 35 30 25
Asp Thr Lys Val Phe Leu Val Pro Arg Gly Gln Ala Ser Leu Arg Phe Val Ala Ala Asp Ser Cys Gly Ile Thr Gln Gln Ala
NeoR/KanR
Asp Thr Lys Val Phe Leu Val Pro Arg Gly Gln Ala Ser Leu Arg Phe Val Ala Ala Asp Ser Cys Gly Ile Thr Gln Gln Ala

CAGTCATAGCCGAATAGCCTCTCCACCCAAGCGGCCGGAGAACCTGCGTGCAATCCATCTTGTTCAATCATGCGAAACGATCCTC 4845
GTCAGTATCGGCTTATCGGAGAGGTGGGTTCGCCGGCCTCTTGGACGCACGTTAGGTAGAACAAGTTAGTACGCTTTGCTAGGAG
20 15 10 5 1
Trp Asp Tyr Gly Phe Leu Arg Glu Val Trp Ala Ala Pro Ser Gly Ala His Leu Gly Asp Gln Glu Ile Met
NeoR/KanR
Trp Asp Tyr Gly Phe Leu Arg Glu Val Trp Ala Ala Pro Ser Gly Ala His Leu Gly Asp Gln Glu Ile Met

ClaI*
BspDI* StuI SfiI
ATCCTGTCTCTTGATCGATCTTTGCAAAAGCCTAGGCCTCCAAAAAAGCCTCCTCACTACTTCTGGAATAGCTCAGAGGCCGAGG 4930
TAGGACAGAGAACTAGCTAGAAACGTTTTCGGATCCGGAGGTTTTTTCGGAGGAGTGATGAAGACCTTATCGAGTCTCCGGCTCC
SV40 promoter
SV40 ori

```
CGGCCTCGGCCTCTGCATAAATAAAAAAAATTAGTCAGCCATGGGGCGGAGAATGGGCGGAACTGGGCGGAGTTAGGGGCGGGAT
                                                                                          5015
GCCGGAGCCGGAGACGTATTTATTTTTTTTAATCAGTCGGTACCCCGCCTCTTACCCGCCTTGACCCGCCTCAATCCCCGCCCTA
<  SV40 promoter
   SV40 ori

GGGCGGAGTTAGGGGCGGGACTATGGTTGCTGACTAATTGAGATGCATGCTTTGCATACTTCTGCCTGCTGGGGAGCCTGGGGAC
                                                                                          5100
CCCGCCTCAATCCCCGCCCTGATACCAACGACTGATTAACTCTACGTACGAAACGTATGAAGACGGACGACCCCTCGGACCCCTG
<  SV40 promoter

          CsiI
          SexAI*
TTTCCACACCTGGTTGCTGACTAATTGAGATGCATGCTTTGCATACTTCTGCCTGCTGGGGAGCCTGGGGACTTTCCACACCCTA
                                                                                          5185
AAAGGTGTGGACCAACGACTGATTAACTCTACGTACGAAACGTATGAAGACGGACGACCCCTCGGACCCCTGAAAGGTGTGGGAT
<  SV40 promoter

ACTGACACACATTCCACAGCTGGTTCTTTCCGCCTCAGGACTCTTCCTTTTTCAATATTATTGAAGCATTTATCAGGGTTATTGT
                                                                                          5270
TGACTGTGTGTAAGGTGTCGACCAAGAAAGGCGGAGTCCTGAGAAGGAAAAAGTTATAATAACTTCGTAAATAGTCCCAATAACA
<  SV40 promoter   <  AmpR promoter

CTCATGAGCGGATACATATTTGAATGTATTTAGAAAAATAAACAAATAGGGGTTCCGCGCACATTTCCCCGAAAAGTGCCACCTG
                                                                                          5355
GAGTACTCGCCTATGTATAAACTTACATAAATCTTTTTATTTGTTTATCCCCAAGGCGCGTGTAAAGGGGCTTTTCACGGTGGAC
<  AmpR promoter

ACGCGCCCTGTAGCGGCGCATTAAGCGCGGCGGGTGTGGTGGTTACGCGCAGCGTGACCGCTACACTTGCCAGCGCCCTAGCGCC
                                                                                          5440
TGCGCGGGACATCGCCGCGTAATTCGCGCCGCCCACACCACCAATGCGCGTCGCACTGGCGATGTGAACGGTCGCGGGATCGCGG
   f1 ori  >

CGCTCCTTTCGCTTTCTTCCCTTCCTTTCTCGCCACGTTCGCCGGCTTTCCCCGTCAAGCTCTAAATCGGGGGCTCCCTTTAGGG
                                                                                          5525
GCGAGGAAAGCGAAAGAAGGGAAGGAAAGAGCGGTGCAAGCGGCCGAAAGGGGCAGTTCGAGATTTAGCCCCCGAGGGAAATCCC
   f1 ori  >

                                                                  DraIII
TTCCGATTTAGTGCTTTACGGCACCTCGACCCCAAAAAACTTGATTAGGGTGATGGTTCACGTAGTGGGCCATCGCCCTGATAGA
                                                                                          5610
AAGGCTAAATCACGAAATGCCGTGGAGCTGGGGTTTTTTGAACTAATCCCACTACCAAGTGCATCACCCGGTAGCGGGACTATCT
   f1 ori  >

CGGTTTTTCGCCCTTTGACGTTGGAGTCCACGTTCTTTAATAGTGGACTCTTGTTCCAAACTGGAACAACACTCAACCCTATCTC
                                                                                          5695
GCCAAAAAGCGGGAAACTGCAACCTCAGGTGCAAGAAATTATCACCTGAGAACAAGGTTTGACCTTGTTGTGAGTTGGGATAGAG
   f1 ori  >

                    PsiI
GGTCTATTCTTTTGATTTATAAGGGATTTTGCCGATTTCGGCCTATTGGTTAAAAAATGAGCTGATTTAACAAAAATTTAACGCG
                                                                                          5780
CCAGATAAGAAAACTAAATATTCCCTAAAACGGCTAAAGCCGGATAACCAATTTTTTACTCGACTAAATTGTTTTTAAATTGCGC
   f1 ori  >

AATTTT   3'
         ...   5786
TTAAAA   5'
f1 ori  >
```

Supplementary Figure S2: Mutant Sequence with Annotated Features

All features and annotations are displayed using SnapGene to illustrate the mutant sequence that was transfected into CHO-K1 plasmids. The only difference between the mutant and the wildtype sequence is *rs2405442:T>C*. Feature annotations comprise the following eight pages.

Sequence: PILRA_mt_Cterminaltag.dna (Circular / 5786 bp)
Enzymes: Unique 6+ Cutters (50 of 678 total)
Features: 18 total

Unique Cutters **Bold**

```
5' AACAAAATATTAACGCTTACAATTTCCATTCGCCATTCAGGCTGCGCAACTGTTGGGAAGGGCGATCGGTGCGGGCCTCT   80
3' TTGTTTTATAATTGCGAATGTTAAAGGTAAGCGGTAAGTCCGACGCGTTGACAACCCTTCCCGCTAGCCACGCCCGGAGA
```

f1 ori

```
TCGCTATTACGCCAGCTGGCGAAAGGGGGATGTGCTGCAAGGCGATTAAGTTGGGTAACGCCAGGGTTTTCCCAGTCACG   160
AGCGATAATGCGGTCGACCGCTTTCCCCCTACACGACGTTCCGCTAATTCAACCCATTGCGGTCCCAAAAGGGTCAGTGC
```

```
ACGTTGTAAAACGACGGCCAGTGCCAAGCTGATCTATACATTGAATCAATATTGGCAATTAGCCATATTAGTCATTGGTT   240
TGCAACATTTTGCTGCCGGTCACGGTTCGACTAGATATGTAACTTAGTTATAACCGTTAATCGGTATAATCAGTAACCAA
```

M13 fwd

BsrGI

```
ATATAGCATAAATCAATATTGGCTATTGGCCATTGCATACGTTGTATCTATATCATAATATGTACATTTATATTGGCTCA   320
TATATCGTATTTAGTTATAACCGATAACCGGTAACGTATGCAACATAGATATAGTATTATACATGTAAATATAACCGAGT
```

SpeI **AseI**

```
TGTCCAATATGACCGCCATGTTGACATTGATTATTGACTAGTTATTAATAGTAATCAATTACGGGGTCATTAGTTCATAG   400
ACAGGTTATACTGGCGGTACAACTGTAACTAATAACTGATCAATAATTATCATTAGTTAATGCCCCAGTAATCAAGTATC
```

CMV enhancer

```
CCCATATATGGAGTTCCGCGTTACATAACTTACGGTAAATGGCCCGCCTGGCTGACCGCCCAACGACCCCCGCCCATTGA   480
GGGTATATACCTCAAGGCGCAATGTATTGAATGCCATTTACCGGGCGGACCGACTGGCGGGTTGCTGGGGGCGGGTAACT
```

CMV enhancer

```
CGTCAATAATGACGTATGTTCCCATAGTAACGCCAATAGGGACTTTCCATTGACGTCAATGGGTGGAGTATTTACGGTAA   560
GCAGTTATTACTGCATACAAGGGTATCATTGCGGTTATCCCTGAAAGGTAACTGCAGTTACCCACCTCATAAATGCCATT
```

CMV enhancer

```
ACTGCCCACTTGGCAGTACATCAAGTGTATCATATGCCAAGTCCGCCCCCTATTGACGTCAATGACGGTAAATGGCCCGC   640
TGACGGGTGAACCGTCATGTAGTTCACATAGTATACGGTTCAGGCGGGGGATAACTGCAGTTACTGCCATTTACCGGGCG
```

CMV enhancer

SnaBI

```
CTGGCATTATGCCCAGTACATGACCTTACGGGACTTTCCTACTTGGCAGTACATCTACGTATTAGTCATCGCTATTACCA   720
GACCGTAATACGGGTCATGTACTGGAATGCCCTGAAAGGATGAACCGTCATGTAGATGCATAATCAGTAGCGATAATGGT
```

CMV enhancer

```
TGGTGATGCGGTTTTGGCAGTACACCAATGGGCGTGGATAGCGGTTTGACTCACGGGGATTTCCAAGTCTCCACCCCATT   800
ACCACTACGCCAAAACCGTCATGTGGTTACCCGCACCTATCGCCAAACTGAGTGCCCCTAAAGGTTCAGAGGTGGGGTAA
```

CMV promoter

CMV enhancer

```
GACGTCAATGGGAGTTTGTTTTGGCACCAAAATCAACGGGACTTTCCAAAATGTCGTAATAACCCCGCCCCGTTGACGCA   880
CTGCAGTTACCCTCAAACAAAACCGTGGTTTTAGTTGCCCTGAAAGGTTTTACAGCATTATTGGGGCGGGGCAACTGCGT
```

CMV promoter

```
AATGGGCGGTAGGCGTGTACGGTGGGAGGTCTATATAAGCAGAGCTCGTTTAGTGAACCGTCAGAATTTTGTAATACGAC   960
TTACCCGCCATCCGCACATGCCACCCTCCAGATATATTCGTCTCGAGCAAATCACTTGGCAGTCTTAAAACATTATGCTG
```

CMV promoter

T7 promoter

EcoRI SalI AccI BamHI Acc65I KpnI BglII AsiSI

TCACTATAGGGCGGCCGGGAATTCGTCGACTGGATCCGGTACCGAGGAGATCTGCCGCCGCGATCGCGCCACCatgggtc 1040

AGTGATATCCCGCCGGCCCTTAAGCAGCTGACCTAGGCCATGGCTCCTCTAGACGGCGGCGCTAGCGCGGTGGtacccag

T7 promoter

PILRA_mt

Kozak sequence

Met Gly

ggcccctgctgctgcccctactgcccctgctgctgccgccagcatttctgcagcctagtggctccacaggatctggtcca 1120

ccggggacgacgacggggatgacggggacgacgacggcggtcgtaaagacgtcggatcaccgaggtgtcctagaccaggt

PILRA_mt

mt_ctg

Arg Pro Leu Leu Leu Pro Leu Leu Pro Leu Leu Leu Pro Pro Ala Phe Leu Gln Pro Ser Gly Ser Thr Gly Ser Gly Pro

agctacctttatggggtcactcaaccaaaacacctctcagcctccatgggtggctctgtggaaatccccttctccttcta 1200

tcgatggaaataccccagtgagttggttttgtggagagtcggaggtacccaccgagacacctttaggggaagaggaagat

PILRA_mt

Ser Tyr Leu Tyr Gly Val Thr Gln Pro Lys His Leu Ser Ala Ser Met Gly Gly Ser Val Glu Ile Pro Phe Ser Phe Tyr

BmgBI

ttacccctgggagttagccacagctcccgacgtgagaatatcctggagacggggccacttccacaggcagtccttctaca 1280

aatggggaccctcaatcggtgtcgagggctgcactcttataggacctctgccccggtgaaggtgtccgtcaggaagatgt

PILRA_mt

Tyr Pro Trp Glu Leu Ala Thr Ala Pro Asp Val Arg Ile Ser Trp Arg Arg Gly His Phe His Arg Gln Ser Phe Tyr

gcacaaggccgccttccattcacaaggattatgtgaaccggctctttctgaactggacagagggtcagaagagcggcttc 1360

cgtgttccggcggaaggtaagtgttcctaatacacttggccgagaaagacttgacctgtctcccagtcttctcgccgaag

PILRA_mt

Ser Thr Arg Pro Pro Ser Ile His Lys Asp Tyr Val Asn Arg Leu Phe Leu Asn Trp Thr Glu Gly Gln Lys Ser Gly Phe

ctcaggatctccaacctgcagaagcaggaccagtctgtgtatttctgccgagttgagctggacacacggagctcagggag 1440

gagtcctagaggttggacgtcttcgtcctggtcagacacataaagacggctcaactcgacctgtgtgcctcgagtccctc

PILRA_mt

Leu Arg Ile Ser Asn Leu Gln Lys Gln Asp Gln Ser Val Tyr Phe Cys Arg Val Glu Leu Asp Thr Arg Ser Ser Gly Arg

gcagcagtggcagtccatcgaggggaccaaactctccatcacccaggctgtcacgaccaccacccagaggcccagcagca 1520

cgtcgtcaccgtcaggtagctcccctggtttgagaggtagtgggtccgacagtgctggtggtgggtctccgggtcgtcgt

PILRA_mt

Gln Gln Trp Gln Ser Ile Glu Gly Thr Lys Leu Ser Ile Thr Gln Ala Val Thr Thr Thr Thr Gln Arg Pro Ser Ser

tgactaccacctggaggctcagtagcacaaccaccacaaccggcctcagggtcacacagggcaaacgacgctcagactct 1600

actgatggtggacctccgagtcatcgtgttggtggtgttggccggagtcccagtgtgtcccgtttgctgcgagtctgaga

PILRA_mt

Met Thr Thr Thr Trp Arg Leu Ser Ser Thr Thr Thr Thr Thr Gly Leu Arg Val Thr Gln Gly Lys Arg Arg Ser Asp Ser

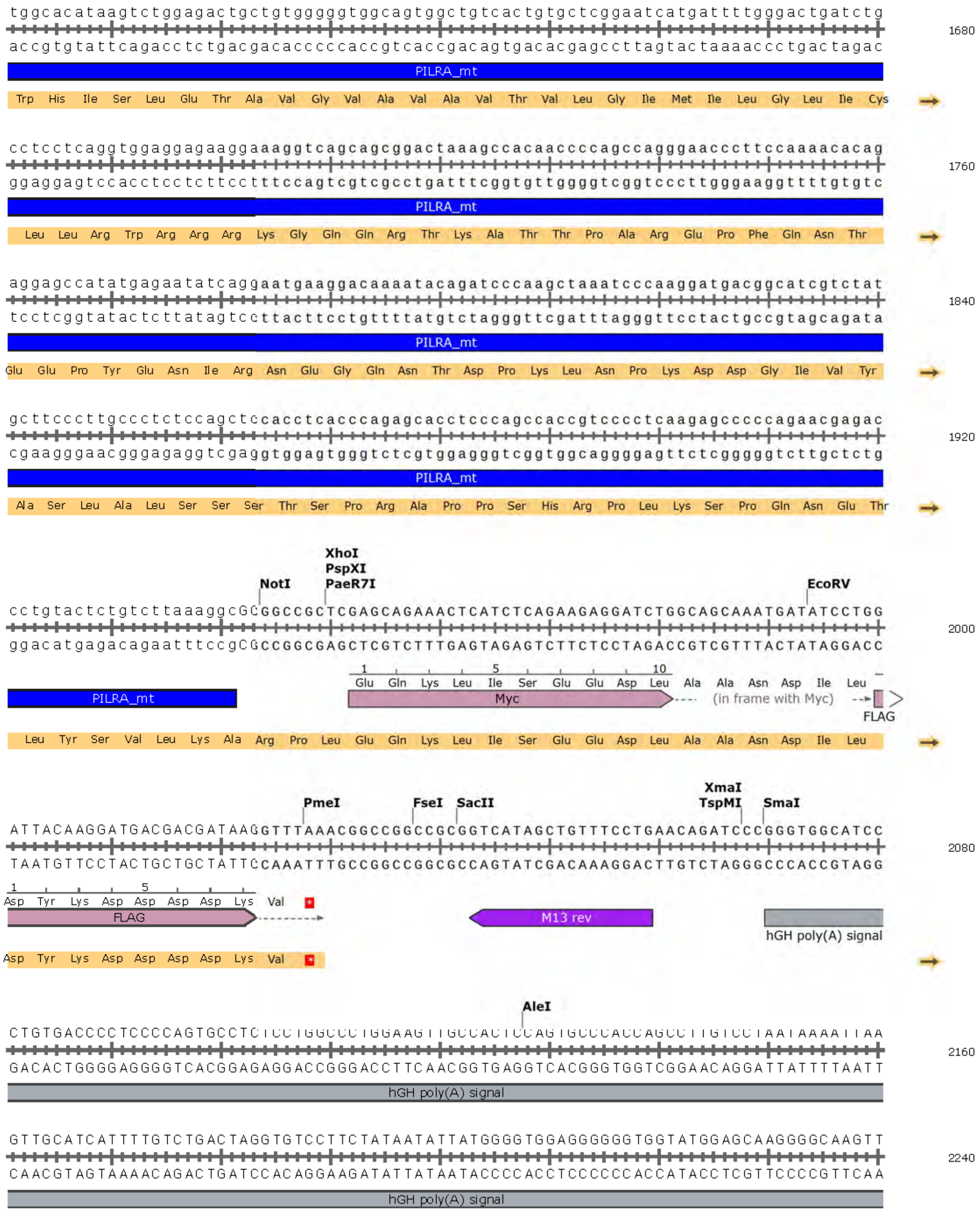
tggcacataagtctggagactgctgtgggggtggcagtggctgtcactgtgctcggaatcatgattttgggactgatctg
accgtgtattcagacctctgacgacacccccaccgtcaccgacagtgacacgagccttagtactaaaaccctgactagac
1680
PILRA_mt
Trp His Ile Ser Leu Glu Thr Ala Val Gly Val Ala Val Ala Val Thr Val Leu Gly Ile Met Ile Leu Gly Leu Ile Cys
cctcctcaggtggaggagaaggaaaggtcagcagcggactaaagccacaaccccagccagggaacccttccaaaacacag
ggaggagtccacctcctcttcctttccagtcgtcgcctgatttcggtgttggggtcggtcccttgggaaggttttgtgtc
1760
PILRA_mt
Leu Leu Arg Trp Arg Arg Arg Lys Gly Gln Gln Arg Thr Lys Ala Thr Thr Pro Ala Arg Glu Pro Phe Gln Asn Thr
aggagccatatgagaatatcaggaatgaaggacaaaatacagatcccaagctaaatcccaaggatgacggcatcgtctat
tcctcggtatactcttatagtccttacttcctgttttatgtctagggttcgatttagggttcctactgccgtagcagata
1840
PILRA_mt
Glu Glu Pro Tyr Glu Asn Ile Arg Asn Glu Gly Gln Asn Thr Asp Pro Lys Leu Asn Pro Lys Asp Asp Gly Ile Val Tyr
gcttcccttgccctctccagctccacctcacccagagcacctcccagccaccgtcccctcaagagcccccagaacgagac
cgaagggaacgggagaggtcgaggtggagtgggtctcgtggagggtcggtggcaggggagttctcgggggtcttgctctg
1920
PILRA_mt
Ala Ser Leu Ala Leu Ser Ser Ser Thr Ser Pro Arg Ala Pro Pro Ser His Arg Pro Leu Lys Ser Pro Gln Asn Glu Thr
XhoI
PspXI
NotI
PaeR7I
EcoRV
cctgtactctgtcttaaaggcGCGGCCGCTCGAGCAGAAACTCATCTCAGAAGAGGATCTGGCAGCAAATGATATCCTGG
ggacatgagacagaatttccgCGCCGGCGAGCTCGTCTTTGAGTAGAGTCTTCTCCTAGACCGTCGTTTACTATAGGACC
2000
1 5 10
Glu Gln Lys Leu Ile Ser Glu Glu Asp Leu Ala Ala Asn Asp Ile Leu
PILRA_mt
Myc
(in frame with Myc)
FLAG
Leu Tyr Ser Val Leu Lys Ala Arg Pro Leu Glu Gln Lys Leu Ile Ser Glu Glu Asp Leu Ala Ala Asn Asp Ile Leu
XmaI
PmeI
FseI
SacII
TspMI
SmaI
ATTACAAGGATGACGACGATAAGGTTTAAACGGCCGGCCGCGGTCATAGCTGTTTCCTGAACAGATCCCGGGTGGCATCC
TAATGTTCCTACTGCTGCTATTCCAAATTTGCCGGCCGGCGCCAGTATCGACAAAGGACTTGTCTAGGGCCCACCGTAGG
2080
1 5
Asp Tyr Lys Asp Asp Asp Asp Lys Val
FLAG
M13 rev
hGH poly(A) signal
Asp Tyr Lys Asp Asp Asp Asp Lys Val
AleI
CTGTGACCCCTCCCCAGTGCCTCTCCTGGCCCTGGAAGTTGCCACTCCAGTGCCCACCAGCCTTGTCCTAATAAAATTAA
GACACTGGGGAGGGGTCACGGAGAGGACCGGGACCTTCAACGGTGAGGTCACGGGTGGTCGGAACAGGATTATTTTAATT
2160
hGH poly(A) signal
GTTGCATCATTTTGTCTGACTAGGTGTCCTTCTATAATATTATGGGGTGGAGGGGGGTGGTATGGAGCAAGGGGCAAGTT
CAACGTAGTAAAACAGACTGATCCACAGGAAGATATTATAATACCCCACCTCCCCCCACCATACCTCGTTCCCCGTTCAA
2240
hGH poly(A) signal

```
        BbsI                                                                 BsgI
GGGAAGACAACCTGTAGGGCCTGCGGGGTCTATTGGGAACCAAGCTGGAGTGCAGTGGCACAATCTTGGCTCACTGCAAT  2320
CCCTTCTGTTGGACATCCCGGACGCCCCAGATAACCCTTGGTTCGACCTCACGTCACCGTGTTAGAACCGAGTGACGTTA
                              hGH poly(A) signal

                                BbvCI                                       BlpI
CTCCGCCTCCTGGGTTCAAGCGATTCTCCTGCCTCAGCCTCCCGAGTTGTTGGGATTCCAGGCATGCATGACCAGGCTCA  2400
GAGGCGGAGGACCCAAGTTCGCTAAGAGGACGGAGTCGGAGGGCTCAACAACCCTAAGGTCCGTACGTACTGGTCCGAGT
                              hGH poly(A) signal

GCTAATTTTTGTTTTTTTGGTAGAGACGGGGTTTCACCATATTGGCCAGGCTGGTCTCCAACTCCTAATCTCAGGTGATC  2480
CGATTAAAAACAAAAAAACCATCTCTGCCCCAAAGTGGTATAACCGGTCCGACCAGAGGTTGAGGATTAGAGTCCACTAG
                              hGH poly(A) signal

                        BstXI
TACCCACCTTGGCCTCCCAAATTGCTGGGATTACAGGCGTGAACCACTGCTCCCTTCCCTGTCCTTCTGATTTTAAAATA  2560
ATGGGTGGAACCGGAGGGTTTAACGACCCTAATGTCCGCACTTGGTGACGAGGGAAGGGACAGGAAGACTAAAATTTTAT
                              hGH poly(A) signal

                                                       AgeI  PflMI
ACTATACCAGCAGGAGGACGTCCAGACACAGCATAGGCTACCTGGCCATGCCCAACCGGTGGGACATTTGAGTTGCTTGC  2640
TGATATGGTCGTCCTCCTGCAGGTCTGTGTCGTATCCGATGGACCGGTACGGGTTGGCCACCCTGTAAACTCAACGAACG
                              hGH poly(A) signal

TTGGCACTGTCCTCTCATGCGTTGGGTCCACTCAGTAGATGCCTGTTGAATTGGGTACGCGGCCAGCGGCGAGCGGTATC  2720
AACCGTGACAGGAGAGTACGCAACCCAGGTGAGTCATCTACGGACAACTTAACCCATGCGCCGGTCGCCGCTCGCCATAG
                 hGH poly(A) signal

                                                             PciI
                                                             AflIII
AGCTCACTCAAAGGCGGTAATACGGTTATCCACAGAATCAGGGGATAACGCAGGAAAGAACATGTGAGCAAAAGGCCAGC  2800
TCGAGTGAGTTTCCGCCATTATGCCAATAGGTGTCTTAGTCCCCTATTGCGTCCTTTCTTGTACACTCGTTTTCCGGTCG

AAAAGGCCAGGAACCGTAAAAAGGCCGCGTTGCTGGCGTTTTTCCATAGGCTCCGCCCCCCTGACGAGCATCACAAAAAT  2880
TTTTCCGGTCCTTGGCATTTTTCCGGCGCAACGACCGCAAAAAGGTATCCGAGGCGGGGGGACTGCTCGTAGTGTTTTTA
                                                      ori

CGACGCTCAAGTCAGAGGTGGCGAAACCCGACAGGACTATAAAGATACCAGGCGTTTCCCCCTGGAAGCTCCCTCGTGCG  2960
GCTGCGAGTTCAGTCTCCACCGCTTTGGGCTGTCCTGATATTTCTATGGTCCGCAAAGGGGGACCTTCGAGGGAGCACGC
                              ori

CTCTCCTGTTCCGACCCTGCCGCTTACCGGATACCTGTCCGCCTTTCTCCCTTCGGGAAGCGTGGCGCTTTCTCATAGCT  3040
GAGAGGACAAGGCTGGGACGGCGAATGGCCTATGGACAGGCGGAAAGAGGGAAGCCCTTCGCACCGCGAAAGAGTATCGA
                              ori

                                                       ApaLI
CACGCTGTAGGTATCTCAGTTCGGTGTAGGTCGTTCGCTCCAAGCTGGGCTGTGTGCACGAACCCCCCGTTCAGCCCGAC  3120
GTGCGACATCCATAGAGTCAAGCCACATCCAGCAAGCGAGGTTCGACCCGACACACGTGCTTGGGGGGCAAGTCGGGCTG
                              ori
```

CGCTGCGCCTTATCCGGTAACTATCGTCTTGAGTCCAACCCGGTAAGACACGACTTATCGCCACTGGCAGCAGCCACTGG 3200
GCGACGCGGAATAGGCCATTGATAGCAGAACTCAGGTTGGGCCATTCTGTGCTGAATAGCGGTGACCGTCGTCGGTGACC
ori

TAACAGGATTAGCAGAGCGAGGTATGTAGGCGGTGCTACAGAGTTCTTGAAGTGGTGGCCTAACTACGGCTACACTAGAA 3280
ATTGTCCTAATCGTCTCGCTCCATACATCCGCCACGATGTCTCAAGAACTTCACCACCGGATTGATGCCGATGTGATCTT
ori

GAACAGTATTTGGTATCTGCGCTCTGCTGAAGCCAGTTACCTTCGGAAAAAGAGTTGGTAGCTCTTGATCCGGCAAACAA 3360
CTTGTCATAAACCATAGACGCGAGACGACTTCGGTCAATGGAAGCCTTTTTCTCAACCATCGAGAACTAGGCCGTTTGTT
ori

ACCACCGCTGGTAGCGGTGGTTTTTTTGTTTGCAAGCAGCAGATTACGCGCAGAAAAAAAGGATCTCAAGAAGATCCTTT 3440
TGGTGGCGACCATCGCCACCAAAAAAACAAACGTTCGTCGTCTAATGCGCGTCTTTTTTTCCTAGAGTTCTTCTAGGAAA
ori

GATCTTTTCTACGGGGTCTGACGCTCAGTGGAACGAAAACTCACGTTAAGGGATTTTGGTCATGAGATTATCAAAAAGGA 3520
CTAGAAAAGATGCCCCAGACTGCGAGTCACCTTGCTTTTGAGTGCAATTCCCTAAAACCAGTACTCTAATAGTTTTTCCT

TCTTCACCTAGATCCTTTTAAATTAAAAATGAAGTTTTAAATCAATCTAAAGTATATATGAGTAACCTGAGGCTATGGCA 3600
AGAAGTGGATCTAGGAAAATTTAATTTTTACTTCAAAATTTAGTTAGATTTCATATATACTCATTGGACTCCGATACCGT

GGGCCTGCCGCCCCGACGTTGGCTGCGAGCCCTGGGCCTTCACCCGAACTTGGGGGGTGGGGTGGGGAAAAGGAAGAAAC 3680
CCCGGACGGCGGGGCTGCAACCGACGCTCGGGACCCGGAAGTGGGCTTGAACCCCCCACCCCACCCCTTTTCCTTCTTTG

GCGGGCGTATTGGCCCCAATGGGGTCTCGGTGGGGTATCGACAGAGTGCCAGCCCTGGGACCGAACCCCGCGTTTATGAA 3760
CGCCCGCATAACCGGGGTTACCCCAGAGCCACCCCATAGCTGTCTCACGGTCGGGACCCTGGCTTGGGGCGCAAATACTT
HSV TK poly(A) signal

CAAACGACCCAACACCGTGCGTTTTATTCTGTCTTTTTATTGCCGTCATAGCGCGGGTTCCTTCCGGTATTGTCTCCTTC 3840
GTTTGCTGGGTTGTGGCACGCAAAATAAGACAGAAAAATAACGGCAGTATCGCGCCCAAGGAAGGCCATAACAGAGGAAG
HSV TK poly(A) signal

CGTGTTTCAGTTAGCCTCCCCCTAGGGTGGGCGAAGAACTCCAGCATGAGATCCCCGCGCTGGAGGATCATCCAGCCGGC 3920
GCACAAAGTCAATCGGAGGGGGATCCCACCCGCTTCTTGAGGTCGTACTCTAGGGGCGCGACCTCCTAGTAGGTCGGCCG

GTCCCGGAAAACGATTCCGAAGCCCAACCTTTCATAGAAGGCGGCGGTGGAATCGAAATCTCGTGATGGCAGGTTGGGCG 4000
CAGGGCCTTTTGCTAAGGCTTCGGGTTGGAAAGTATCTTCCGCCGCCACCTTAGCTTTAGAGCACTACCGTCCAACCCGC

BstBI
TCGCTTGGTCGGTCATTTCGAACCCCAGAGTCCCGCTCAGAAGAACTCGTCAAGAAGGCGATAGAAGGCGATGCGCTGCG 4080
AGCGAACCAGCCAGTAAAGCTTGGGGTCTCAGGGCGAGTCTTCTTGAGCAGTTCTTCCGCTATCTTCCGCTACGCGACGC
265 260 255
* Phe Phe Glu Asp Leu Leu Arg Tyr Phe Ala Ile Arg Gln Ser
NeoR/KanR
* Phe Phe Glu Asp Leu Leu Arg Tyr Phe Ala Ile Arg Gln Ser

AATCGGGAGCGGCGATACCGTAAAGCACGAGGAAGCGGTCAGCCCATTCGCCGCCAAGCTCTTCAGCAATATCACGGGTA 4160
TTAGCCCTCGCCGCTATGGCATTTCGTGCTCCTTCGCCAGTCGGGTAAGCGGCGGTTCGAGAAGTCGTTATAGTGCCCAT
250 245 240 235 230 225
Asp Pro Ala Ala Ile Gly Tyr Leu Val Leu Phe Arg Asp Ala Trp Glu Gly Gly Leu Glu Glu Ala Ile Asp Arg Thr
NeoR/KanR
Asp Pro Ala Ala Ile Gly Tyr Leu Val Leu Phe Arg Asp Ala Trp Glu Gly Gly Leu Glu Glu Ala Ile Asp Arg Thr

GCCAACGCTATGTCCTGATAGCGATCCGCCACACCCAGCCGGCCACAGTCGATGAATCCAGAAAAGCGGCCATTTTCCAC 4240
CGGTTGCGATACAGGACTATCGCTAGGCGGTGTGGGTCGGCCGGTGTCAGCTACTTAGGTCTTTTCGCCGGTAAAAGGTG
220 215 210 205 200
Ala Leu Ala Ile Asp Gln Tyr Arg Asp Ala Val Gly Leu Arg Gly Cys Asp Ile Phe Gly Ser Phe Arg Gly Asn Glu Val
NeoR/KanR
Ala Leu Ala Ile Asp Gln Tyr Arg Asp Ala Val Gly Leu Arg Gly Cys Asp Ile Phe Gly Ser Phe Arg Gly Asn Glu Val

CATGATATTCGGCAAGCAGGCATCGCCATGGGTCACGACGAGATCCTCGCCGTCGGGCATGCTCGCCTTGAGCCTGGCGA 4320
GTACTATAAGCCGTTCGTCCGTAGCGGTACCCAGTGCTGCTCTAGGAGCGGCAGCCCGTACGAGCGGAACTCGGACCGCT
195 190 185 180 175
Met Ile Asn Pro Leu Cys Ala Asp Gly His Thr Val Val Leu Asp Glu Gly Asp Pro Met Ser Ala Lys Leu Arg Ala Phe
NeoR/KanR
Met Ile Asn Pro Leu Cys Ala Asp Gly His Thr Val Val Leu Asp Glu Gly Asp Pro Met Ser Ala Lys Leu Arg Ala Phe

ACAGTTCGGCTGGCGCGAGCCCCTGATGCTCTTCGTCCAGATCATCCTGATCGACAAGACCGGCTTCCATCCGAGTACGT 4400
TGTCAAGCCGACCGCGCTCGGGGACTACGAGAAGCAGGTCTAGTAGGACTAGCTGTTCTGGCCGAAGGTAGGCTCATGCA
170 165 160 155 150 145
Leu Glu Ala Pro Ala Leu Gly Gln His Glu Glu Asp Leu Asp Asp Gln Asp Val Leu Gly Ala Glu Met Arg Thr Arg
NeoR/KanR
Leu Glu Ala Pro Ala Leu Gly Gln His Glu Glu Asp Leu Asp Asp Gln Asp Val Leu Gly Ala Glu Met Arg Thr Arg

GCTCGCTCGATGCGATGTTTCGCTTGGTGGTCGAATGGGCAGGTAGCCGGATCAAGCGTATGCAGCCGCCGCATTGCATC 4480
CGAGCGAGCTACGCTACAAAGCGAACCACCAGCTTACCCGTCCATCGGCCTAGTTCGCATACGTCGGCGGCGTAACGTAG
140 135 130 125 120
Ala Arg Glu Ile Arg His Lys Ala Gln His Asp Phe Pro Cys Thr Ala Pro Asp Leu Thr His Leu Arg Arg Met Ala Asp
NeoR/KanR
Ala Arg Glu Ile Arg His Lys Ala Gln His Asp Phe Pro Cys Thr Ala Pro Asp Leu Thr His Leu Arg Arg Met Ala Asp

AGCCATGATGGATACTTTCTCGGCAGGAGCAAGGTGAGATGACAGGAGATCCTGCCCCGGCACTTCGCCCAATAGCAGCC 4560
TCGGTACTACCTATGAAAGAGCCGTCCTCGTTCCACTCTACTGTCCTCTAGGACGGGGCCGTGAAGCGGGTTATCGTCGG
115 110 105 100 95
Ala Met Ile Ser Val Lys Glu Ala Pro Ala Leu His Ser Ser Leu Leu Asp Gln Gly Pro Val Glu Gly Leu Leu Leu Trp
NeoR/KanR
Ala Met Ile Ser Val Lys Glu Ala Pro Ala Leu His Ser Ser Leu Leu Asp Gln Gly Pro Val Glu Gly Leu Leu Leu Trp

PflFI
Tth111I

AGTCCCTTCCCGCTTCAGTGACAACGTCGAGCACAGCTGCGCAAGGAACGCCCGTCGTGGCCAGCCACGATAGCCGCGCT 4640
TCAGGGAAGGGCGAAGTCACTGTTGCAGCTCGTGTCGACGCGTTCCTTGCGGGCAGCACCGGTCGGTGCTATCGGCGCGA
90 85 80 75 70 65
Asp Arg Gly Ala Glu Thr Val Val Asp Leu Val Ala Ala Cys Pro Val Gly Thr Thr Ala Leu Trp Ser Leu Arg Ala
NeoR/KanR
Asp Arg Gly Ala Glu Thr Val Val Asp Leu Val Ala Ala Cys Pro Val Gly Thr Thr Ala Leu Trp Ser Leu Arg Ala

KasI NarI SfoI PluTI

```
GCCTCGTCTTGCAGTTCATTCAGGGCACCGGACAGGTCGGTCTTGACAAAAAGAACCGGGCGCCCCTGCGCTGACAGCCG   4720
CGGAGCAGAACGTCAAGTAAGTCCCGTGGCCTGTCCAGCCAGAACTGTTTTTCTTGGCCCGCGGGGACGCGACTGTCGGC
```

60 55 50 45 40
Ala Glu Asp Gln Leu Glu Asn Leu Ala Gly Ser Leu Asp Thr Lys Val Phe Leu Val Pro Arg Gly Gln Ala Ser Leu Arg
NeoR/KanR
Ala Glu Asp Gln Leu Glu Asn Leu Ala Gly Ser Leu Asp Thr Lys Val Phe Leu Val Pro Arg Gly Gln Ala Ser Leu Arg

```
GAACACGGCGGCATCAGAGCAGCCGATTGTCTGTTGTGCCCAGTCATAGCCGAATAGCCTCTCCACCCAAGCGGCCGGAG   4800
CTTGTGCCGCCGTAGTCTCGTCGGCTAACAGACAACACGGGTCAGTATCGGCTTATCGGAGAGGTGGGTTCGCCGGCCTC
```

35 30 25 20 15
Phe Val Ala Ala Asp Ser Cys Gly Ile Thr Gln Gln Ala Trp Asp Tyr Gly Phe Leu Arg Glu Val Trp Ala Ala Pro Ser
NeoR/KanR
Phe Val Ala Ala Asp Ser Cys Gly Ile Thr Gln Gln Ala Trp Asp Tyr Gly Phe Leu Arg Glu Val Trp Ala Ala Pro Ser

ClaI*
BspDI*

```
AACCTGCGTGCAATCCATCTTGTTCAATCATGCGAAACGATCCTCATCCTGTCTCTTGATCGATCTTTGCAAAAGCCTAG   4880
TTGGACGCACGTTAGGTAGAACAAGTTAGTACGCTTTGCTAGGAGTAGGACAGAGAACTAGCTAGAAACGTTTTCGGATC
```

10 5 1
Gly Ala His Leu Gly Asp Gln Glu Ile Met
NeoR/KanR
SV40 promoter
SV40 ori
Gly Ala His Leu Gly Asp Gln Glu Ile Met

StuI SfiI

```
GCCTCCAAAAAAGCCTCCTCACTACTTCTGGAATAGCTCAGAGGCCGAGGCGGCCTCGGCCTCTGCATAAATAAAAAAAA   4960
CGGAGGTTTTTTCGGAGGAGTGATGAAGACCTTATCGAGTCTCCGGCTCCGCCGGAGCCGGAGACGTATTTATTTTTTTT
```

SV40 promoter
SV40 ori

```
TTAGTCAGCCATGGGGCGGAGAATGGGCGGAACTGGGCGGAGTTAGGGGCGGGATGGGCGGAGTTAGGGGCGGGACTATG   5040
AATCAGTCGGTACCCCGCCTCTTACCCGCCTTGACCCGCCTCAATCCCCGCCCTACCCGCCTCAATCCCCGCCCTGATAC
```

SV40 promoter
SV40 ori

CsiI
SexAI*

```
GTTGCTGACTAATTGAGATGCATGCTTTGCATACTTCTGCCTGCTGGGGAGCCTGGGGACTTTCCACACCTGGTTGCTGA   5120
CAACGACTGATTAACTCTACGTACGAAACGTATGAAGACGGACGACCCCTCGGACCCCTGAAAGGTGTGGACCAACGACT
```

SV40 promoter

```
CTAATTGAGATGCATGCTTTGCATACTTCTGCCTGCTGGGGAGCCTGGGGACTTTCCACACCCTAACTGACACACATTCC   5200
GATTAACTCTACGTACGAAACGTATGAAGACGGACGACCCCTCGGACCCCTGAAAGGTGTGGGATTGACTGTGTGTAAGG
```

SV40 promoter

```
ACAGCTGGTTCTTTCCGCCTCAGGACTCTTCCTTTTTCAATATTATTGAAGCATTTATCAGGGTTATTGTCTCATGAGCG   5280
TGTCGACCAAGAAAGGCGGAGTCCTGAGAAGGAAAAAGTTATAATAACTTCGTAAATAGTCCCAATAACAGAGTACTCGC
```

SV40 promoter
AmpR promoter

GATACATATTTGAATGTATTTAGAAAAATAAACAAATAGGGGTTCCGCGCACATTTCCCCGAAAAGTGCCACCTGACGCG 5360
CTATGTATAAACTTACATAAATCTTTTTATTTGTTTATCCCCAAGGCGCGTGTAAAGGGGCTTTTCACGGTGGACTGCGC
AmpR promoter
f1 ori

CCCTGTAGCGGCGCATTAAGCGCGGCGGGTGTGGTGGTTACGCGCAGCGTGACCGCTACACTTGCCAGCGCCCTAGCGCC 5440
GGGACATCGCCGCGTAATTCGCGCCGCCCACACCACCAATGCGCGTCGCACTGGCGATGTGAACGGTCGCGGGATCGCGG
f1 ori

CGCTCCTTTCGCTTTCTTCCCTTCCTTTCTCGCCACGTTCGCCGGCTTTCCCCGTCAAGCTCTAAATCGGGGGCTCCCTT 5520
GCGAGGAAAGCGAAAGAAGGGAAGGAAAGAGCGGTGCAAGCGGCCGAAAGGGGCAGTTCGAGATTTAGCCCCCGAGGGAA
f1 ori

DraIII
TAGGGTTCCGATTTAGTGCTTTACGGCACCTCGACCCCAAAAAACTTGATTAGGGTGATGGTTCACGTAGTGGGCCATCG 5600
ATCCCAAGGCTAAATCACGAAATGCCGTGGAGCTGGGGTTTTTTGAACTAATCCCACTACCAAGTGCATCACCCGGTAGC
f1 ori

CCCTGATAGACGGTTTTTCGCCCTTTGACGTTGGAGTCCACGTTCTTTAATAGTGGACTCTTGTTCCAAACTGGAACAAC 5680
GGGACTATCTGCCAAAAAGCGGGAAACTGCAACCTCAGGTGCAAGAAATTATCACCTGAGAACAAGGTTTGACCTTGTTG
f1 ori

PsiI
ACTCAACCCTATCTCGGTCTATTCTTTTGATTTATAAGGGATTTTGCCGATTTCGGCCTATTGGTTAAAAAATGAGCTGA 5760
TGAGTTGGGATAGAGCCAGATAAGAAAACTAAATATTCCCTAAAACGGCTAAAGCCGGATAACCAATTTTTTACTCGACT
f1 ori

TTTAACAAAAATTTAACGCGAATTTT 3′
... 5786
AAATTGTTTTTAAATTGCGCTTAAAA 5′
f1 ori
